# The IICR (inverse instantaneous coalescence rate) as a summary of genomic diversity: insights into demographic inference and model choice

**DOI:** 10.1038/s41437-017-0005-6

**Published:** 2017-11-08

**Authors:** Lounès Chikhi, Willy Rodríguez, Simona Grusea, Patrícia Santos, Simon Boitard, Olivier Mazet

**Affiliations:** 1 CNRS, Université Paul Sabatier, ENFA, UMR 5174 EDB (Laboratoire Évolution & Diversité Biologique), Bât. 4R1, F-31062 Toulouse, France; 2 Université de Toulouse, UPS, EDB, F-31062 Toulouse, France; 3 Instituto Gulbenkian de Ciência, Rua da Quinta Grande, No. 6, P-2780-156 Oeiras, Portugal; 4 Université de Toulouse, Institut National des Sciences Appliquées, Institut de Mathématiques de Toulouse, F-31077 Toulouse, France; 5 GenPhySE, Université de Toulouse, INRA, INPT, INP-ENVT, Castanet Tolosan, France

## Abstract

Several inferential methods using genomic data have been proposed to quantify and date population size changes in the history of species. At the same time an increasing number of studies have shown that population structure can generate spurious signals of population size change. Recently, Mazet et al. (2016) introduced, for a sample size of two, a time-dependent parameter, which they called the IICR (inverse instantaneous coalescence rate). The IICR is equivalent to a population size in panmictic models, but not necessarily in structured models. It is characterised by a temporal trajectory that suggests population size changes, as a function of the sampling scheme, even when the total population size was constant. Here, we extend the work of Mazet et al. (2016) by (i) showing how the IICR can be computed for any demographic model of interest, under the coalescent, (ii) applying this approach to models of population structure (1D and 2D stepping stone, split models, two- and three-island asymmetric gene flow, continent-island models), (iii) stressing the importance of the sampling strategy in generating different histories, (iv) arguing that IICR plots can be seen as summaries of genomic information that can thus be used for model choice or model exclusion (v) applying this approach to the question of admixture between humans and Neanderthals. Altogether these results are potentially important given that the widely used PSMC (pairwise sequentially Markovian coalescent) method of Li and Durbin (2011) estimates the IICR of the sample, not necessarily the history of the populations.

## Introduction

The habitats of most animal and plant species have expanded and contracted on various occasions during their history (Hewitt 2000). In the last forty years, population geneticists have been increasingly interested in understanding and describing the specific signatures of such expansions and contractions in the genome of extant or extinct species (Nei et al. 1975; Tajima 1989; Slatkin and Hudson 1991; Rogers and Harpending 1992). It seems indeed reasonable to assume that during the periods of spatial habitat expansion the total population size increased, whereas during the contraction periods, it decreased. Whereas initial studies suggested or assumed that there was a relatively simple relationship between population size changes inferred from genetic data and real demographic changes, it has become increasingly clear that the relationship is not necessarily simple (Marjoram and Donnelly 1994; Wakeley 1999; Mazet et al. 2016). For instance, a growing number of studies have shown that population structure *per se* can generate spurious signals of population size change, even when populations were stationary and that these signals depend on the sampling strategy used (Wakeley 1999, 2001; Beaumont 2004; Nielsen and Beaumont 2009; Städler et al. 2009; Chikhi et al. 2010; Peter et al. 2010; Heller et al. 2013; Paz-Vinas et al. 2013; Mazet et al. 2015, 2016). In addition, several studies have shown that the direction of change inferred from genetic data can even be opposite to the actual change in total size when populations are structured (Wakeley 1999; Mazet et al. 2016). The fact that genetic data can lead us to infer demographic changes that may never have taken place is potentially problematic and still not fully understood or taken into account. There is, therefore, a need to understand whether and how spurious population size changes may be generated under different models of population structure.

To do that we use the IICR (inverse instantaneous coalescence rate) introduced by Mazet et al. (2016) for a sample of size two (i.e., two haploid genomes or one diploid individual). In a few words, the IICR is, as its name indicates, the inverse of the rate at which coalescence events take place for a given sample configuration under a specific demographic model, as a function of time. Since the coalescence rate is inversely proportional to the population size in a simple Wright-Fisher model (Hudson et al. 1990), it seems reasonable to interpret the IICR as a population size. However, Mazet et al. (2016) stressed that if the IICR is equivalent to a population size in panmictic models it can actually be misleading in structured models. For instance, they showed that in an n-island model (Wright 1931) where the total size stays constant the IICR actually exhibits the trend of a decreasing population. They also found that in the case in which all demes increase in size instantaneously the IICR can identify this increase but found also that the opposite trend of a declining population will dominate the IICR, for the parameter values tested. In other words, the IICR can be, under some conditions at least, more influenced by the structure of the population than by the change in total size. Given that the IICR curves can be estimated by the pairwise sequentially Markovian coalescent (PSMC) method of Li and Durbin (2011) and that this method has been widely used (Groenen et al. 2012; Zhan et al. 2013; Zhao et al. 2013; Fitak et al. 2016), it is important to understand the properties of the IICR for a sample of size two under models of population structure beyond the n-island model. This would allow population geneticists to better understand and interpret the plots inferred by the PSMC or other similar methods such as the MSMC (multiple sequentially Markovian coalescent), diCal (demographic inference using composite approximate likelihood), stairway plot or PopSizeABC (Schiffels and Durbin 2013; Sheehan et al. 2013; Liu and Fu 2015; Boitard et al. 2016).

In the present study we (i) extend the work of Mazet et al. (2016) by expliciting how the IICR can be obtained for any demographic model for which coalescence times (*T*_2_) can be simulated and by providing a python script to compute and plot the IICR (Supplementary Fig. S1), (ii) apply this approach to several models of population genetics and predict the IICR plots for these models and different sampling schemes, (iii) show that the different IICR curves can be seen as a summaries of genomic diversity and thus as “summary statistics” that could be used in an approximate Bayesian computation framework, (iv) show that the IICR plots can thus be used for model exclusion, (v) apply this approach, as a proof of concept, to a published study investigating admixture between Neanderthals and humans. We find that the models used cannot explain the PSMC observed in humans or Neanderthals. These models should thus probably not be used to argue for or against admixture.

## Definition and computation of the IICR

### Theory: definition and interpretation of the IICR

The IICR was introduced by Mazet et al. (2016) in a study in which they explored the properties of $f_{T_2}(t)$, the probability density function (*pdf*) of coalescence times for a sample of size 2 (*T*_2_), where *t* is measured in units of the haploid population size, *N* (see below). They showed that the IICR is a function of time and, more specifically, that $IICR(t) = {\frac{{{\Bbb P}\left( {T_2  >t} \right)}}{{f_{T_2}(t)}}}$. While the IICR was explicitly defined as a function of time we follow Mazet et al. (2016) and drop the time parameter for the sake of notation simplicity in the rest of the manuscript. Also, we focused only on the results obtained for a sample of size two, it was thus not necessary to make the sample size explicit in the present manuscript.

**Fig. 1:**
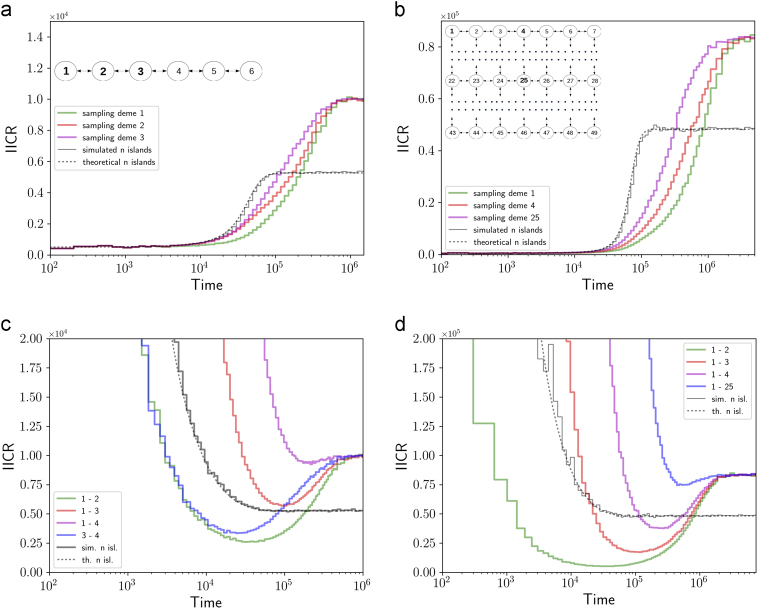
Inferred population size changes for 1D and 2D stepping stone models with constant size. In panels **a**, **b** the two genes were sampled in the same deme, whereas in panels **c, d** they were sampled in different demes. Panels **a, c** show the results for the 1D stepping stone, whereas panels **b**, **d** show the 2D stepping stone model results. For comparison we also plotted the results for a comparable n-island model (same number of islands and *M* = 1) in all panels, using the same simulation approach (black ragged solid line) together with the theoretical (exact) IICR (black continuous solid line). Panels **a**, **b** contain a simplified representation of the models. In addition each panel contains a legend for the coloured lines. For instance, in panel **a** the two genes were obtained in demes 1 (green line), 2 (red line) and 3 (magenta line)

Since the IICR is the inverse of a coalescence rate it is natural to interpret it as a proxy for population size (Wakeley 2001; Sjödin et al. 2005; Wakeley and Sargsyan 2009; Li and Durbin 2011). However, in models with population structure the IICR is characterised by a time-dependent trajectory rather than a single value, even when the actual total size does not vary. The IICR of two genes can be derived analytically for any model for which the distribution of coalescence times, *T*_2_, is known. Following the seminal work of Herbots (1994) and Wilkinson-Herbots (1998) on $f_{T_2}(t)$, Mazet et al. (2016) derived the IICR for the *n*-island model, where there are *n* islands (or demes) of constant haploid size *N*, connected by gene flow with a migration rate *m*, where *M* = 2*Nm* and *M* is the number of immigrants (genes) in each island every generation. The IICR for two genes sampled in the *same* deme (the *IICR*_s_) or in *different* demes (*IICR*_d_) can be expressed as functions of *n* and *M* (Mazet et al. 2016 and Supplementary files). The *IICR*_s_ and *IICR*_d_ exhibited different trajectories, namely a history of monotonic S-shaped decrease for the *IICR*_s_ and an L-shaped history of recent expansion for the *IICR*_d_ (Figs. 1 and 2 in Mazet et al. 2016). This shows the importance of sampling on demographic inference as several authors have stressed before (Wakeley 1999; Beaumont 2004; Städler et al. 2009; 117 Chikhi et al. 2010; Heller et al. 2013; Paz-Vinas et al. 2013). Due to the importance of the sampling scheme, the *IICR*_s_ and *IICR*_d_ terminology, will be used when necessary.

**Fig. 2:**
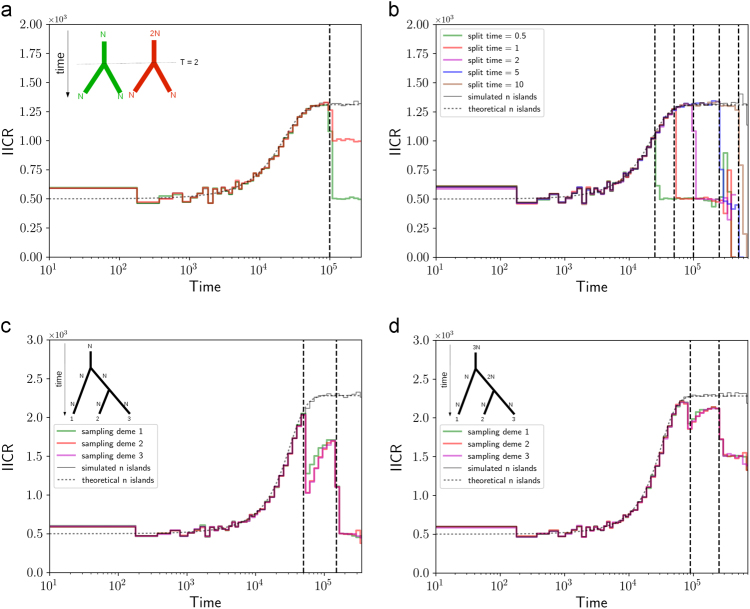
*IICR*_s_ for population split models. Inferred population size changes for population split models with one or two splitting events. In the four panels the two genes were sampled in the same deme. Panel **a** shows a model where one ancestral population of size 2*N* (red line) or *N* (green line) splits in two populations of size *N* at time *T* = 2. In panel **b** the ancestral population of size *N* splits in two populations of size *N* at different times. Panels **c**, **d** show a split with a three-island model and an ancestral population of size *N* (panel **c**) and 3*N* (panel **d**) at time *T* = 1 and 3. In all models the populations exchange genes at a rate *M* = 1 after the split. For comparison we also plotted the simulation and theoretical curves for a comparable n-island model (black lines)

Before going through the properties and computation of the IICR plots, we try to clarify why Mazet et al. (2016) introduced this concept, and why they did not call it an instantaneous effective size, even though this would have seemed simpler and more natural. To do that we make use of the n-island model and ask what its population size is. A straightforward answer is that the total population size, *N*_*T*_, is simply the product *N*_*T*_ = *nN*. This answer is perfectly valid and can be seen as the “real” population size, since this is the actual total number of haploid genomes. This answer is, however, unsatistactory to most population geneticists since the amount of genetic diversity that can be maintained in an n-island is different from that of a panmictic population of size *nN*. The concept of effective size, *N*_*e*_, allows us to account for that, and *N*_*e*_ is typically a function of *n*, *N* and *m*, *N*_*e*_ = *f*(*n*, *N*, *m*), since the migration rate will play a role in how diversity is maintained in the set of *n* populations. For instance, the nucleotide diversity effective size of Nei and Takahata (1993) for the n-island model is $N_e = N\left( {n + \frac{{\left( {n - 1} \right)^2}}{{nM}}} \right)$. The implicit assumption of the *N*_*e*_ concept is that a single number is sufficient to summarise some property of the complex model it represents (Sjödin et al. 2005).

This interpretation is, however, not fully consistent with the observation that population structure generates signals of population size change (Storz and Beaumont 2002; Beaumont 2004; Städler et al. 2009; Chikhi et al. 2010; Peter et al. 2010). By definition a population size change requires a model with at least two different *N*_*e*_ values. This shows that some coalescent-related property of structured models cannot be summarised by one *N*_*e*_. A more critical issue is that the IICR is a function of the sampling scheme considered. To our knowledge this is not a property of any known definition of *N*_*e*_. In other words, the concept of effective size is useful but potentially confusing or problematic. It provides additional information over the simple count of genes in the population and helps explain diversity patterns as many studies have shown. However, it can miss important properties of the coalescent. Previous authors have noted that the computation of *N*_*e*_ may be misleading about the actual changes in size of real populations (*N*_*T*_) under models where migration rates change (e.g., Wakeley 1999; Mazet et al. 2016).

We believe that the IICR, by avoiding the notion of effective size, but focusing on what is actually measured or estimated, solves many of these problems. Indeed, the IICR incorporates the notion that coalescence rates can vary as a function of the sampling strategy. It is important because these apparent changes in population size are *constitutive* of the model and sample (location and timing). In other words, if we sampled individuals in the past (assuming that population structure did not change) the IICR would simply be shifted towards the past. Even if the shape of the IICR suggested that the instantaneous *N*_*e*_ was larger in the past, a sample from that time point would not exhibit more diversity (since we assumed a stationary model). And that sample would have the same IICR except that it would start at that sample’s time point. It would thus be shifted towards the past (Supplementary Fig. S2). In the case of a real population size change, the ancient sample should exhibit a higher or lower diversity depending on its instantaneous *N*_*e*_ at that generation compared to the instantaneous *N*_*e*_ at time zero. In a structured population, the notion of instantaneous *N*_*e*_ as estimated from the IICR is thus misleading (Supplementary Fig. S2). The concept of instantaneous *N*_*e*_ thus misrepresents this crucial property of the IICR. In short, the n-island model does not have an *N*_*e*_, valid for all parameter values, it has several IICR curves that shift and change with the time of sampling and sampling scheme. It is the combination of these IICR curves that can be seen as a signature of that specific model.

There are, however, connections with classical theory. For instance, the asymptotic value of the *IICR*_s_ for large *t* values becomes *N*/*β*, where

$$\beta = \frac{1}{2}\left( {1 + \frac{n}{{n - 1}}M - \sqrt {\left( {1 + \frac{n}{{n - 1}}M} \right)^2 - \frac{{4M}}{{n - 1}}} } \right).$$

When $M \gg 1$, *N*/*β* simplifies and is approximately equal to the *N*_*e*_ of Nei and Takahata (1993). This *N*_*e*_ computation thus implicitly assumes that the S-shaped decrease from the plateau can be neglected, and that the S-shaped trajectory can be replaced by a horizontal line, corresponding to the *N*/*β* plateau. It is true that when $M \gg 1$ the IICR plots are mainly influenced by this asymptotic value (Mazet et al. 2016). But when *M* is large, the effect of population structure decreases and the formula will be closer to *nN*. In other words, the *N*_*e*_ approximation of the IICR above is most appropriate for cases where population structure is less relevant. When the migration rate is low $\left( {M \ll 1} \right)$, that is, when we need to understand the effects of population structure, this formula will produce high *N*_*e*_ values close to *N*/*β*, whereas the IICR trajectory will reflect the influence of the local deme size and to a much lesser extent that of the plateau. Note that when *M* is small *N*/*β* tends towards *N*(*n* − 1)/*M*. For instance, if there are *n* = 10 islands of size *N* = 1000 connected by *M* = 0.1 migrants per generation, the ancient plateau will be close to 90,000 even though the total population size is *N*_*T*_ = 10,000. The IICR of such a model would thus suggest a drastic decrease from an ancient population size of *N* = 90,000 to *N* = 1000. This is rather different from a scenario of constant *N*_*e*_ = 90,000, implicit in the *N*_*e*_ calculation. The *N*_*e*_ approximation thus ignores the most recent part of the IICR, which, with low *M* values, can actually extend quite far back in the past.

### Methods: IICR computation

It is possible to estimate the IICR by simulating independent *T*_2_ values and using them to estimate the IICR at various time points *t*_*i*_, as follows (Mazet et al. 2016):

1 $$\widehat {IICR(t_i)} = \frac{{1 - \widehat {F_{T_2}(t_i)}}}{{\widehat {f_{T_2}\left( {t_i} \right)}}}$$

where $\widehat {F_{T_2}\left( {t_i} \right)}$ is the estimated or empirical cumulative distribution function of *T*_2_ and $\widehat {f_{T_2}\left( {t_i} \right)}$ is an estimated approximation of its density around *t*_*i*_.

We can thus predict the IICR for any model of interest for which *T*_2_ values can be simulated. It can then be compared to the PSMC results by simulating genomic data under the same demographic model and sampling scheme. In the next section, we identify several models commonly used in population genetics and provide the IICR curves for them. In all cases data were simulated using the *ms* software (Hudson 2002). For each scenario 10^6^ independent *T*_2_ values were simulated and the IICR computed and plotted with a python script available at: https://github.com/willyrv/IICREstimator. Details of the models and all *ms* commands are provided in the Supplementary files. In most cases the figures were scaled so that the deme size was *N* = 500, and assuming a generation time of 25 years. Finally, for the scaling with human genomic data we used a mutation rate of 2.5 × 10^−8^, as in Li and Durbin (2011). The exact values of these parameters are to some extent of limited importance as we are mainly interested in general principles.

## Results: predicting the IICR for several population genetics models

### Stepping stone models

Figure 1 shows the *IICR*_s_ and *IICR*_d_ curves for a 1D (panels 1a, 1c) and a 2D (panels 1b, 1d) stepping stone model. In both cases the IICR curves of a comparable n-island model with the same number of demes are also represented for comparison. The figure shows that, for both the 1D and 2D stepping stone models, the *IICR*_s_ indicates an S-shaped population decrease from a large and ancient population whose size depends on both *n* and *M* (Supplementary Material), to a small population whose current size is *N*, the size of a deme. Compared to the n-island model both the 1D and 2D stepping stone models plateau (going backward in time) at values higher than *N*/*β*. This is in agreement with the intuition and theoretical results (Rousset 2004), suggesting that stepping stone models are “more structured” than n-island models, and should thus have a larger *N*_*e*_ (or *N*/*β*). This trend was observed for all *n* and *M* values tested (Supplementary Material).

We also found that, in the 1D model, and going backwards in time, the *IICR*_*s*_ quickly becomes very large as the number of islands increases. For instance, for *M* = 1 and *n* = 15 we could not observe the plateau of the *IICR*_s_ (going backwards) based on the values of *T*_2_ (Supplementary Figs). Another striking result of 1D and 2D models is that the location of the sample (panels 1a, b) influences the shape of the IICR even if the IICR curves converge towards the same values in both the recent and ancient past. Qualitatively, the results of the change in sampling scheme are similar between the 1D (Fig. 1a) and 2D (Fig. 1b) stepping stone models. The relative positions of the three coloured lines are similar. The green lines (deme 1 in the 1D and 2D stepping stone models) always indicate a population decrease that started and finished more recently than the red or magenta (edge or central samples) lines.

When we sample haploid genomes in two different demes (this is equivalent to analysing the local offspring of a first generation migrant) we uncover a signal of recent population growth for the n-island model. However, there is a major difference compared to the n-island model as the locations of the two genes can generate more complex demographies. When genes were sampled in distant demes we observed a simple monotonic increase (as in the n-island model), but when genes were sampled in neighbouring demes we observed a more complex demographic history where a bottleneck can be seen in the recent past, just before the recent expansion (1c and 1d).

Altogether these differences between 1D and 2D stepping stone models and the n-island model, suggest that it may be possible to identify properties that a model should have (structure vs. no structure, spatial structure vs. no spatial structure, etc.) to explain a particular set of PSMC plots obtained from several individuals. Note that the two plateaus in panel 1a are identical to those in panel 1c. The IICR plots may thus differ as a function of the sampling scheme (in the same or different demes) but the plateau is the same. A similar point can be made for panels 1b, d.

### Population split models

Figure 2a shows the *IICR*_s_ curves for a model where one ancestral population of size 2*N* (red line) or *N* (green line) splits in two populations of size *N* at time *T* = 2. After the split the two populations exchange genes at a rate *M* = 1. The first splitting model (red line) assumes that the total population remains constant whereas the second splitting model (green line) assumes an instantaneous doubling of the size during the splitting event. The green and red lines show the *IICR*_s_ for a diploid individual sampled in one of the two daughter populations. As expected, between the present and *T* (*i.e*., for *t* < *T*) the *IICR*_s_ is identical to that of the n-island model for *n* = 2 and *M* = 1 (thin black line). Then, as we go backward in time and reach *t* = *T*, the *IICR*_s_ drops and plateaus at the value corresponding to the size of the ancestral population, because in a panmictic population the IICR is equal to *N*_*T*_, the size of the population. Thus, forward in time, a population split at *T* = 2 will be interpreted as a population increase followed by a collapse, even though the model actually corresponds to either a constant or a doubling of *N*_*T*_, the total population size.

Now, to stress the difference between the IICR and the *N*_*e*_ interpretation of population structure we could plot the changes in *N*_*e*_ that would be predicted under a classical interpretation. In the ancient past the ancestral population is panmictic and the three interpretations (*N*_*T*_, IICR and *N*_*e*_) are identical and equal to the total population size. We thus have *N*_*e*_ = *N* or *N*_*e*_ = 2*N*, depending on the split model assumed. Between *t* = *T* and *t* = 0, the two daughter populations behave as a two-island model and we can apply the formula of Nei and Takahata (1993) $N_e = N\left( {n + \frac{{(n - 1)^2}}{{nM}}} \right) = 2.5N$. The splitting model will thus be seen as a simple stepwise population increase from *N*_*e*_ = *N* to *N*_*e*_ = 2.5*N* (or from *N*_*e*_ = 2*N* to *N*_*e*_ = 2.5*N*). Thus, the three points of view produce three rather different interpretations. From the point of view of the total number of genes there was no population size change in one model or a doubling in the other, whereas from an *N*_*e*_ point of view there always was a stepwise increase. Both views are different from the IICR plots, which correspond to what the PSMC method would infer. The IICR also integrates the fact that the sampling scheme is important. Depending on where the samples were obtained a different history (the IICR) will be inferred and will be perfectly legitimate, but should not be interpreted here in terms of population size change.

Figure 2b shows similar plots for the case where the ancestral population is of size *N* but with *T* = 0.5, 1, 2, 5, 10. This panel shows that the size of the “hump” depends on *T*, the timing of the splitting event, and on the shape of the IICR for the corresponding two-island model at *t* = *T*, which itself depends on *M* and on the size of the ancestral population. In these scenarios *N*_*e*_ always exhibits the stepwise increase mentioned above but at different timings. In the special case where the two populations split and are not connected by gene flow, it is straightforward to infer the *IICR*_s_. In a model where the ancestral population was of size *N* the *IICR*_s_ indicates a constant size since from the viewpoint of the sampled genes the population size is always equal to *N*. In the case where the ancestral population size is equal to 2*N* the distribution of coalescence times will depend on *N* in the recent past (for *t* < *T*) and then on 2*N*. The *IICR*_s_ will thus identify forward in time a reduction in size by a factor two. Thus, even in the case where there is no gene flow, a population size change is detected with the *IICR*_s_ when there is none (*N*_*T*_ = *N*), whereas none is detected by the *IICR*_*s*_ or PSMC, when there is one (*N*_*T*_ doubles). In this scenario the *N*_*e*_ interpretation would be either that *N*_*e*_ = *N*, since the population is completely isolated, or *N*_*e*_ = ∞, when the set of two populations is considered.

In the case of a split with three populations it is more difficult to identify general properties as several parameters play a role in shaping the final IICR curves beside the sampling scheme. In panels 2c, d and for the parameters chosen here, the *IICR*_s_ identifies (forward in time) two periods of population size increase followed by population size decrease, which, again, never took place since population sizes were either constant or increasing in a stepwise manner during the splitting events.

### Asymmetrical gene flow and continent-island models

**Fig. 3:**
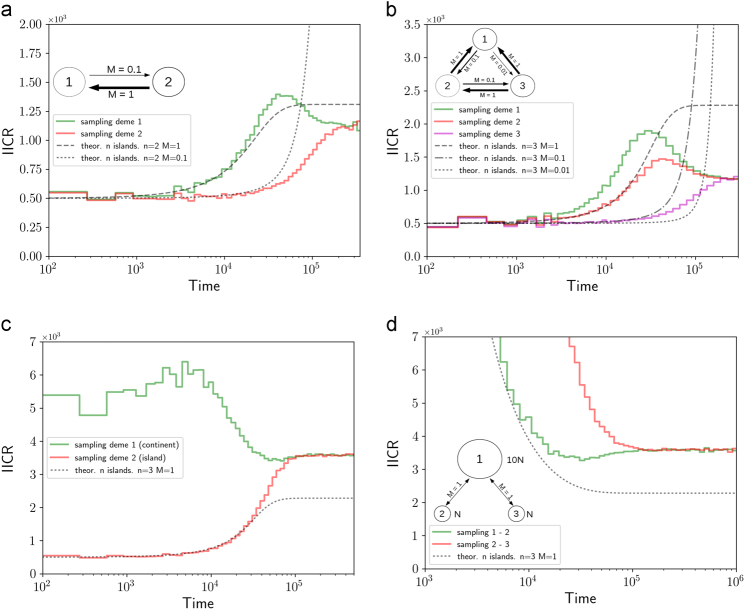
*IICR*_s_ for asymmetrical gene flow and continent-island models. In the first three panels the two genes were sampled in the same deme, whereas in panel **d** they were sampled in two different demes. Panels **a**, **b** show a model with two or three islands, respectively, that exchange genes at different rates (*M*_12_ = 1, *M*_21_ = 0.1, *M*_23_ = 1, *M*_32_ = 0.1, *M*_21_ = 0.1, *M*_31_ = 0.01 and *M*_13_ = 1, where *M*_*ij*_ is the number of haploid genomes in deme *i* that migrated in from deme *j*. Panel **c** shows the result for the continent-island model with a size ratio 1:10 (the continent is ten times larger than the island) and *M* = 1. For comparison we also plotted the simulation and theoretical results for a comparable n-island model in all panels, as in the other figures. For panels **a**, **b** we added the theoretical IICR for *M* = 0.1 and *M* = 0.01 since these are the rates of exchange between some of the demes

Figure 3 shows the IICR under models with two or three islands characterised by asymmetrical migration rates. In the two-island model we assume that migration in one direction is ten times greater than in the other direction. This is represented in panel 3a. A model where the migration rate is one hundred times greater in one direction than in the other exhibits the same qualitative pattern and is represented in the Supplementary Materials. In panel 3b a model with three islands and asymetrical gene flow is used. The green line corresponds to the case where the island that receives more migrants is sampled (deme 1 in both panels), whereas the red (panel 3a) and magenta (panel 3b) lines correspond to the case where the island that sends more migrants was sampled (deme 2 in panel 3a and deme 3 in panel 3b). Interestingly, when an island receives more than it sends migrants, it exhibits an IICR with a hump that would be interpreted, forward in time, as a history in which the population grew in size and then decreased until the present. In the three-island case two islands exhibit humps, and the less notable hump is observed for island 2. It is difficult to interpret these humps, but it is as if the arrival of divergent alleles in an island connected to a continent generated an influx of diversity that would be interpreted as an expansion signal.

In the case of the continent-island model simulated here (Fig. 3c, d), where the continent is ten times larger than the island, the two *IICR*_s_ curves start in the recent time at different values that reflect the local deme size, as expected. The green curve corresponds to a diploid sampled in the continent, whereas the red curve corresponds to a diploid sampled in the island. The *IICR*_s_ curves join in the ancient past at a level that is surprisingly lower than the size of the continent. This means that, forward in time genomic data from a diploid sampled in the continent could tell the history of a population that continuously and monotonically increased in size, whereas an individual sampled in an island would suggest a history of continuous and monotonic S-shaped decrease.

It is worth stressing again that in all these models that involve an asymmetry in size or migration rates, the total population size, and the migration rates were constant, and none of the islands changed in size.

### Application to real data

In the previous sections we have argued that the IICR curves for *T*_2_ are informative about genomic diversity under specific demographic models and sampling strategies. They can thus be seen as a summaries of genomic information providing sample-dependent information on the demographic history of the species of interest. We have focused on simple, and not so simple, population genetics models for two main reasons. First, they are often used to make statements or inference about the demographic history of species (see below). Second, even though they are simple, they can generate complex IICR plots that resemble the PSMC plots obtained for various species. What we argue is that for any species for which a PSMC plot can be obtained, and for which some demographic models has been used to infer, say, splitting times or admixture proportions, it would seem reasonable to validate these demographic models by determining whether their IICR plots are compatible with the PSMC plots. If they differ significantly this suggests that the models cannot reproduce major summaries of genomic information, and other models should be investigated too. Here, we show how this can be done with real data and then discuss some consequences and perspectives in the final section.

**Fig. 4:**
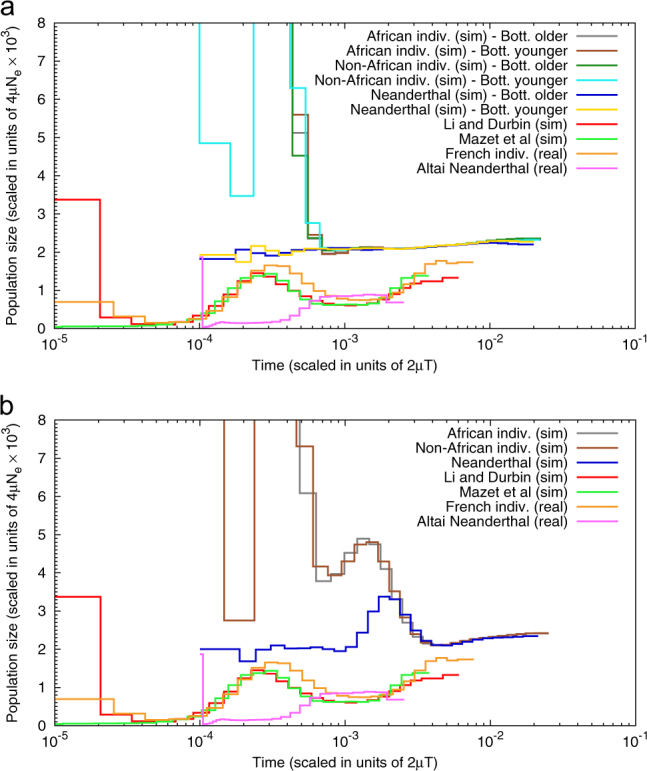
PSMC plots for the admixture and ancient structure models of Yang et al. (2012). This figure shows the PSMC plots for genomic data simulated under the models of admixture (panel **a**) and ancient population structure (panel **b**). The models of Yang et al. (2012) have three populations corresponding to Africans, non-Africans (Europeans or Asians) and Neanderthals. We obtained the PSMC plots for one individual from each of these populations, with parameter values used by the authors. Additional figures can be found in the Supplementary Materials. Here, we represent individuals simulated under the three main models, namely recent admixture with a bottleneck that is either (i) younger, or (ii) older than the admixure event and, (iii) ancient structure. In panel **a** the African, Non-African and Neanderthal individuals were simulated under the model of recent admixture with a bottleneck that was either older or younger than the admixture event. In panel **b** the PSMCs were obtained for an African, Non-African and Neanderthal individual simulated under the model of ancient structure. For the simulated Neanderthals the PSMC curves stop in the past at a time corresponding to the age of the real Neanderthal sample (pink line). The PSMC results obtained for the real genomic data of a French (human, isolated orange line) and a Neanderthal (unknown nationality, pink line) are represented for comparison. The red and light green lines that are on top of each other represent the PSMC plots based on the *ms* commands of Li and Durbin (2011) and Mazet et al. (2016), respectively

To demonstrate how our approach can be used with genomic data we identified a study (Yang et al. 2012), which aimed at determining whether the presence of “Neanderthal DNA” that has been reported in some human populations was due to admixture between Neanderthals and humans or to ancient population structure without admixture. Yang et al. (2012) noted that previous studies had been unable to separate two simple models, one with admixture and one without admixture, because they were using the D statistic of Green et al. (2010). They suggested to use a conditioned site frequency spectrum (*dcfs* for doubly conditioned frequency spectrum) to determine whether it would identify differences missed by the D statistic. Yang et al. (2012) used *ms* commands to generate genetic data under the models of interest and found that the ancient structure model used could not produce *dcfs* resembling those observed in real data, whereas the admixture model could. They concluded that the admixture model was better and that the ancient structure model should be rejected. Since then this study has been cited to support models of admixture between humans and Neanderthals but it has also been criticised on the basis that other models of population structure might still explain present-day data without admixture (Eriksson and Manica 2012). Here, we take this study as a proof of concept regarding the potential use of the *IICR*_s_ curves as summaries of genomic diversity. As our Fig. 4 shows, we found that none of the models used in that study can actually explain the PSMC plots of human populations (see also the Supplementary Materials). In their study Yang et al. (2012) were thorough and used various combinations of parameters for both the ancient structure and admixture models. Between the models, different IICR plots are generated, some of which are flat while others generate large population expansion signals. The family of models that seems to produce small humps that could (but still do not) resemble those observed in human PSMC plots is that of the ancient structure models, not the admixture models. The fact that the ancient structure model only produces one hump (instead of two in real data) and that its temporal location is at odds with the human PSMC plots humps suggests that the ancient structure model chosen by Yang et al. (2012) is unlikely to capture the complexity of human demographic history. These results do not mean that admixture is rejected. It only means that the models used in this study cannot be used as a case for or against admixture between humans and Neanderthals. It is noteworthy that the simulated *dcfs* could fit observed genomic data, despite the fact that the corresponding IICR curves are different. This suggests that the *dcfs* and the IICR plots capture different types of genomic information and should perhaps be used together. Our results are in agreement with Eriksson and Manica (2012) who showed that other models of population structure can in principle explain shared polymorphism between Neanderthals and modern humans without admixture. More work is probably needed to determine whether population structure can explain all aspects of genomic information and to solve this controversial issue.

## Discussion

### The *IICR*_s_ and *IICR*_d_ as summaries of genome-wide information

By definition of the IICR, a panmictic model with the appropriate changes in population size will always perfectly explain a particular set of *T*_2_ values, even if the latter were actually generated under some unknown non panmictic model (Mazet et al. 2016). While the PSMC and MSMC methods of Li and Durbin (2011) and Schiffels and Durbin (2013), are probably the best methods available to infer the IICR, the results of these methods should be interpreted with care when the populations under study are likely to be structured.

In a panmictic model all individuals are exchangeable and should thus all exhibit the same IICR (and PSMC) plot. If different individuals exhibit different PSMC plots this would thus suggest that some level of population structure is present and may require caution. In addition, in models with population structure there may be significant differences between the *IICR*_s_ and *IICR*_d_. Under the n-island model, the IICR of a hybrid individual (*IICR*_d_) exhibits a spurious signal of recent population expansion. This is not expected in a model of total panmixia where there is no clear way to define the *IICR*_s_ and *IICR*_d_ since “hybrid” individuals are to a large extent meaningless. Thus, the IICR contains information to separate panmixia from population structure, by simply using haploid genomes from one or several locations (Mazet et al. 2016).

Here, we extended the work of Mazet et al. (2016) to several models. We found that for the 1D and 2D stepping stone models, the *IICR*_s_ exhibits a monotonic S-shaped decrease whereas the *IICR*_d_ exhibits a monotonic increase when genes are sampled in distant demes and a decrease followed by an expansion when they are sampled in neighbouring demes. Thus, the addition of space or isolation by distance added a level of complexity because demes are not exchangeable. As a consequence there can only be two types of IICR plots in the n-island model, one *IICR*_s_ and one *IICR*_d_, whereas the 1D and 2D stepping stone models exhibit various *IICR*_s_ and *IICR*_d_ plots depending on the way genomes are sampled. It is thus theoretically possible to separate a model of population structure that accounts for space from a model that does not. This could be done by analysing individuals from different locations and generating artificially admixed genomes using individuals from neighbouring and distant demes.

This interest for the properties coalescence rates across individuals can also be seen in the notion of cross coalescence rates of Schiffels and Durbin (2013), that several authors have used to improve their understanding of the demographic history of species. We should also mention the work of Kim et al. (2015) who were also interested in the distribution of coalescent times in models of population size change and independently describe an algorithm that computes the bounds of inference of the IICR. These authors, however, were mainly interested in models of population size change and estimating bounds in population size changes. Similarly, Kishida (2017) simulated genomic data under a split model for whales and then inferred the corresponding PSMC plots, which he compared to the observed PSMC for minke whales. These studies show that various authors have been reaching similar ideas starting from different point of views, and echoeing the seminal studies of Wakeley (1999) and Marjoram and Donnelly (1994) on population structure and demography.

### The IICR provides a solid framework to interpret human and other PSMC plots

As a proof of concept, let us now take the PSMC plots that have been obtained for humans from different regions of the world. Going forward in time, these plots typically show two humps that are shared by all individuals sampled to date. In the more recent past the plots for individuals sampled in different continents start to diverge and seem to either reach different plateaus or to exhibit a recent rapid population increase. This increase could be related to the global increase in human population sizes during the Neolithic transition, or could be due to the variance in estimates of coalescence rates by the PSMC method. The fact that these plots significantly differ in the recent past confirms the trivial fact that humans from different continents do not come from a single panmictic population, but the plots also show that the different locations sampled cannot be considered as isolated populations. As a consequence, interpretations in terms of population size changes should be taken with caution, and a model of population structure seems thus necessary to fully understand human genetic diversity.

Mazet et al. (2016) showed that a simple n-island model with only three changes in migration rates was enough to explain existing PSMC plots in humans without changes in population size. The only population size change required was in the recent past to model the Neolithic expansion. Their objective was to discuss the limitations of panmictic models and they did not defend their model as the best model. Indeed, the n-island model cannot capture the differences between regions. This is why we explored stepping stone models here. The results of the stepping stone models suggest that they can indeed explain differences among regions to some extent. However, the PSMC plots from humans sampled in different continents differ in their recent past by the different levels at which the IICR appears to stabilise before the Neolithic expansion. This thus suggests that one needs a modified stepping stone model with demes of different sizes.

Similarly, caution is required to interpret the part where the PSMC plots are identical in the ancient past. Here, we saw that a plateau in the ancient past does not mean that individuals sampled today derive from one big “ancestral” panmictic population. The plateau that IICR plots exhibit for several demographic models is expected as a consequence of the statistical properties of the coalescent. Plateaus appear for all the models studied here even when there was no large panmictic ancestral population. For complex models involving population structure and variable migration rates Mazet et al. (2016) noted that IICR plots similar to human PSMC plots, with two humps can be obtained. This means that different individuals sampled in different demes would see their IICR converge in the ancient past even though the model never assumes a single “ancestral” population.

One important result of the n-island model and that was confirmed here for other models is that, forward in time, the IICR always tends towards the size of the deme where the genomes were sampled in. This means that under structured models the recent decrease seen in many PSMC plots is constitutive, and not necessarily meaningful in terms of population size change. It is thus informative about local deme size in the region that was sampled. And the slope of the decrease is informative about the rate of gene flow and the type of structure. The 1D and 2D stepping stone models assume that all demes have the same size and thus that all IICR plots converge to the same local deme size (Fig. 1). We can thus suggest that, to explain human PSMC plots, we will need models that are spatially explicit, and where deme size varies in space. Exploring such models is beyond the scope of this study but would be extremely important.

### Explaining humps and bumps in PSMC plots: connectivity, changes in connectivity, population splits, asymmetry and sampling schemes

The humps that are typically observed in PSMC plots for many species can be explained either by actual changes in total population size (i.e., changes in coalescence rates are directly related to changes in *N*_*T*_ or *N*_*e*_) or by changes in the level of connection between populations (i.e., changes in coalescence rates are due to connectivity and changes in connectivity and sampling). When populations are structured, changes in *N*_*T*_ do not necessarily produce the expected humps. Changes in connectivity might thus explain some PSMC plots without the need to invoke major population size changes (Mazet et al. 2016). Here, by exploring population split and asymmetrical two- or three-island models, we found that humps and bumps that are not related in a simple manner to actual changes in total population size can also be generated. In split models humps seem to be related to the splitting times but in ways that are different from the trajectories of both *N*_*T*_ and *N*_*e*_. This suggests that population split models are potentially good models to explain genomic data in various species. This may also indirectly explain their success. However, this does not mean that they will necessarily provide meaningful insights into the evolution of species and populations. Tree models ignore space and may thus miss important aspects of genomic diversity.

In the asymmetric island models explored here, a hump was observed in the absence of any change in the structure of the model (there was no change in size or connectivity). This is a new and fascinating result because it suggests that it is the asymmetry between islands that generates the humps.

Another issue is that of the sampling scheme. We found for nearly all models explored here that the *IICR*_s_ could significantly vary depending on the deme sampled, and in ways that are not always trivial to interpret. In the asymmetrical island models it was when we sampled two genes from the island that received more gene flow that the *IICR*_s_ detected a population increase, whereas two genes sampled in the other island produced an S-shaped *IICR*_s_ that resembled those of a classical n-island model. The importance of the sampling scheme for demographic inference has been stressed by various authors (Wakeley 1999; Städler et al. 2009; Chikhi et al. 2010; Heller et al. 2013; Paz-Vinas et al. 2013; Mazet et al. 2016). Here we suggest that the *IICR*_s_ will vary and additionally that these differences should be used for inference. If different individuals from the same species exhibit different PSMC plots, then models should be explored that account for population structure, spatial structure, asymmetry, etc. Currently such differences would likely be seen as the result of actual differences in the demographic histories of the sampled populations. We showed here that this is not necessarily the case.

### Conclusion and perspectives

Altogether our results showed that *IICR*_s_ and *IICR*_d_ plots can be seen as summaries of genomic information whose shapes inform us on the models that may or not be useful to explain specific PSMC plots observed in real data. We focused on a handful of models that are often used in the literature but the same approach should be used to explore more models. For all the models studied, there are conditions under which the IICR curves identify spurious population size changes. Also, when some asymmetry exists humps can be generated without any temporal change of parameters. Additionally, the fact that the IICR plots of different individuals converge in the ancient past should not be equated to panmixia in a mythical ancestral population from which modern populations derive. For several models we restricted our analysis to equilibrium conditions. It would be important to study these models under non-equilibrium conditions.

We also showed that it is possible to use our framework to look at real data. This is possible because the PSMC method allows reconstructing the IICR from genomic data. In the case of the study by Yang et al. (2012) we found that none of the models used could explain the human or Neanderthal PSMC plots (Fig. 4), something that the authors could not have integrated at the time. Our results suggest that statements about admixture between humans and Neanderthals should take advantage of as much genomic information as possible. The results presented here do not rule out admixture (as observed in the *Oase* sample). However, before ancient population structure is ruled out, it should be evaluated in all its complexity.

While ignoring population structure may be potentially misleading, it is not always clear whether a structured model should be used and whether it will provide more sensible results. Indeed, population size changes have likely taken place during expansions and contractions in parallel and possibly in relation to changes in migration patterns. Separating the contributions of changes in population size from changes in gene flow patterns in present-day patterns of genomic diversity may be one of the greatest challenges facing population geneticists. Our study provides only one way towards this general direction, by quickly and simply identifying or excluding models that cannot explain the data. The distribution of coalescence times and the IICR plots can be seen as new and easy ways to summarise genomic diversity, and could both be used within an ABC framework (Beaumont et al. 2002).

Throughout this study we focused on the IICR as defined for *T*_2_. It is straightforward to extend the concept to larger sample sizes and obtain the IICR for any *T*_*k*_, where *k* > 2 is the number of haploid genomes, as long as the corresponding *T*_*k*_ values can be simulated. This would allow us to compare the results of the MSMC and of the PSMC under different models and sampling schemes.

The simulation approach advocated here is powerful because it allows to study models for which no analytical results are available. However, it also has its limitations since it does not provide a full mathematical understanding of the properties of the IICR (see Mazet et al. (2015) for a similar point). For instance, we cannot generalise our results beyond the range of parameter values tested here. Another avenue of research would be to determine analytically or computationally the distribution of the *T*_*k*_. This is difficult for *k* > =2 for most models integrating population structure. In a study in progress we (S. Grusea, W. Rodriguez, D. Pinchon, L. Chikhi, S. Boitard, O. Mazet) have studied the properties of $T_2^3$, which corresponds *T*_2_ assuming that *T*_3_ has already occurred. This can be done for the n-island model and for the model of population size change that has exactly the same *T*_3_ distribution as the n-island model. We find that the models can be differentiated with the $T_2^3$ distributions. Thus, there is hope that some analytical results can be derived, and that progress is just ahead.

## Electronic supplementary material

supplementary material

## References

[CR1] Beaumont M (2004). Recent developments in genetic data analysis: what can they tell us about human demographic history?. Heredity.

[CR2] Beaumont MA, Zhang W, Balding DJ (2002). Approximate Bayesian computation in population genetics. Genetics.

[CR3] Boitard S, Rodriguez W, Jay F, Mona S, Austerlitz F (2016). Inferring population size history from large samples of genome-wide molecular data-an approximate bayesian computation approach. PLoS Genet.

[CR4] Chikhi L, Sousa VC, Luisi P, Goossens B, Beaumont MA (2010). The confounding effects of population structure, genetic diversity and the sampling scheme on the detection and quantification of population size changes. Genetics.

[CR5] Eriksson A, Manica A (2012). Effect of ancient population structure on the degree of polymorphism shared between modern human populations and ancient hominins. Proc Natl Acad Sci.

[CR6] Fitak RR, Mohandesan E, Corander J, Burger PA (2016). The de novo genome assembly and annotation of a female domestic dromedary of north african origin. Mol Ecol Resour.

[CR7] Green RE, Krause J, Briggs AW, Maricic T, Stenzel U, Kircher M, Patterson N, Li H, Zhai W, Fritz MHY (2010). A draft sequence of the neandertal genome. Science.

[CR8] Groenen M, Archibald A, Uenishi H, Tuggle C, Takeuchi Y, Rothschild M, Rogel-Gaillard C, Park C, Milan D, Megens H, Li S, Larkin D, Kim H, Frantz L, Caccamo M, Ahn H, Aken B, Anselmo A, Anthon C, Auvil L, Badaoui B, Beattie C, Bendixen C, Berman D, Blecha F, Blomberg J, Bolund L, Bosse M, Botti S, Bujie Z (2012). Analyses of pig genomes provide insight into porcine demography and evolution. Nature.

[CR9] Heller R, Chikhi L, Siegismund HR (2013). The confounding effect of population structure on Bayesian skyline plot inferences of demographic history. PLoS ONE.

[CR10] Herbots HMJD (1994) Stochastic models in population genetics: genealogy and genetic differentiation in structured populations. PhD thesis. Queen Mary University of London: London

[CR11] Hewitt G (2000). The genetic legacy of the Quaternary ice ages. Nature.

[CR12] Hudson RR (2002). Generating samples under a Wright–Fisher neutral model of genetic variation. Bioinformatics.

[CR13] Hudson RR (1990). Gene genealogies and the coalescent process. Oxford Surv Evol Biol.

[CR14] Kim J, Mossel E, Rácz MZ, Ross N (2015). Can one hear the shape of a population history?. Theor Popul Biol.

[CR15] Kishida T (2016). Population history of antarctic and common minke whales inferred from individual whole-genome sequences. Mar Mamm Sci.

[CR16] Li H, Durbin R (2011). Inference of human population history from individual whole-genome sequences. Nature.

[CR17] Liu X, Fu Y-X (2015). Exploring population size changes using SNP frequency spectra. Nat Genet.

[CR18] Marjoram P, Donnelly P (1994). Pairwise comparisons of mitochondrial dna sequences in subdivided populations and implications for early human evolution. Genetics.

[CR19] Mazet O, Rodríguez W, Chikhi L (2015). Demographic inference using genetic data from a single individual: Separating population size variation from population structure. Theor Popul Biol.

[CR20] Mazet O, Rodriguez W, Grusea S, Boitard S, Chikhi L (2016). On the importance of being structured: instantaneous coalescence rates and human evolution—lessons for ancestral population size inference&quest. Heredity.

[CR21] Nei M, Maruyama T, Chakraborty R (1975). The bottleneck effect and genetic variability in populations. Evolution.

[CR22] Nei M, Takahata N (1993). Effective population size, genetic diversity, and coalescence time in subdivided populations. J Mol Evol.

[CR23] Nielsen R, Beaumont MA (2009). Statistical inferences in phylogeography. Mol Ecol.

[CR24] Paz-Vinas I, Quéméré E, Chikhi L, Loot G, Blanchet S (2013). The demographic history of populations experiencing asymmetric gene flow: combining simulated and empirical data. Mol Ecol.

[CR25] Peter BM, Wegmann D, Excoffier L (2010). Distinguishing between population bottleneck and population subdivision by a Bayesian model choice procedure. Mol Ecol.

[CR26] Rogers AR, Harpending H (1992). Population growth makes waves in the distribution of pairwise genetic differences. Mol Biol Evol.

[CR27] Rousset, F (2004) Genetic structure and selection in subdivided populations (MPB-40). Princeton University Press: Monographs in Population Biology-40

[CR28] Schiffels S, Durbin R (2013). Inferring human population size and separation history from multiple genome sequences. Nat Genet.

[CR29] Sheehan S, Harris K, Song YS (2013). Estimating variable effective population sizes from multiple genomes: a sequentially markov conditional sampling distribution approach. Genetics.

[CR30] Sjödin P, Kaj I, Krone S, Lascoux M, Nordborg M (2005). On the meaning and existence of an effective population size. Genetics.

[CR31] Slatkin M, Hudson RR (1991). Pairwise comparisons of mitochondrial DNA sequences in stable and exponentially growing populations. Genetics.

[CR32] Städler T, Haubold B, Merino C, Stephan W, Pfaffelhuber P (2009). The impact of sampling schemes on the site frequency spectrum in nonequilibrium subdivided populations. Genetics.

[CR33] Storz JF, Beaumont MA (2002). Testing for genetic evidence of population expansion and contraction: an empirical analysis of microsatellite DNA variation using a hierarchical Bayesian model. Evolution.

[CR34] Tajima F (1989). The effect of change in population size on DNA polymorphism. Genetics.

[CR35] Wakeley J (1999). Nonequilibrium migration in human history. Genetics.

[CR36] Wakeley J (2001). The coalescent in an island model of population subdivision with variation among demes. Theor Popul Biol.

[CR37] Wakeley J, Sargsyan O (2009). Extensions of the coalescent effective population size. Genetics.

[CR38] Wilkinson-Herbots HM (1998). Genealogy and subpopulation differentiation under various models of population structure. J Math Biol.

[CR39] Wright S (1931). Evolution in Mendelian populations. Genetics.

[CR40] Yang MA, Malaspinas AS, Durand EY, Slatkin M (2012). Ancient structure in Africa unlikely to explain Neanderthal and non-African genetic similarity. Mol Biol Evol.

[CR41] Zhan X, Pan S, Wang J, Dixon A, He J, Muller MG, Ni P, Hu L, Liu Y, Hou H (2013). Peregrine and saker falcon genome sequences provide insights into evolution of a predatory lifestyle. Nat Genet.

[CR42] Zhao S, Zheng P, Dong S, Zhan X, Wu Q, Guo X, Hu Y, He W, Zhang S, Fan W (2013). Whole-genome sequencing of giant pandas provides insights into demographic history and local adaptation. Nat Genet.

