# Supplementary material for: The IICR (inverse instantaneous coalescence rate) as a summary of genomic diversity: insights into demographic inference and model choice
Source: Heredity (Edinb). 2017 Nov 8;120(1):13–24. doi: 10.1038/s41437-017-0005-6 (PMC5837117; doi:10.1038/s41437-017-0005-6)
Supplement: Supplementary file 1 — supplementary material [file 41437_2017_5_MOESM1_ESM.pdf]

The IICR (inverse instantaneous coalescence rate) as a summary of genomic diversity: insights into demographic inference and model choice. Supplementary Materials.

Chikhi Lounès, Rodríguez Willy, Grusea Simona, Santos Patrícia, Boitard Simon, Mazet Olivier

July 7, 2017

1 Definition and computation of the IICR

The IICR was introduced by Mazet *et al.* (2016) in a study in which they explored the properties of the distribution of coalescence times for a sample of size two (T_2) under different sampling schemes. In Mazet *et al.* (2016) and in the present manuscript we explained why the IICR should not be interpreted as a population size unless population structure can be rejected or neglected. In models with population structure the IICR is characterized by a time-dependent and sample-dependent trajectory rather than a single value, even when the actual size does not vary. This means that there isn't one demographic history but an inferred history that depends on the sampling scheme. For a sample size of two, the different IICRs represent thus different perspectives of the same "meta-population history". These different IICRs can be derived analytically for any model for which the distribution of coalescence times, T_2, is known. As an example we reproduce here the $IICR_s$ and $IICR_d$ for the n-island model, where we have a set of n islands (or demes) of constant haploid size N, connected by gene flow with a migration rate m, where $M = 4Nm$ and M is the number of immigrants (genes) in each island every generation. For two genes sampled in the *same* deme the $IICR_s$ can be expressed as a function of n and M:

$$IICR_s(t) = \frac{\mathbb{P}(T_2 > t)}{f_{T_2^s}^{n-island}(t)} = \frac{\frac{a}{\alpha}e^{-\alpha t} + \frac{1-a}{\beta}e^{-\beta t}}{ae^{-\alpha t} + (1-a)e^{-\beta t}} = \frac{(1-\beta)e^{-\alpha t} + (\alpha - 1)e^{-\beta t}}{(\alpha - \gamma)e^{-\alpha t} + (\gamma - \beta)e^{-\beta t}} \quad (1)$$

where $f_{T_2^s}^{n-island}(t)$ is the probability density function (*pdf*) of the coalescence time T_2 of two genes sampled in the same deme, (here t is expressed in units of $2N$ generations), and where

$$a = \frac{\gamma - \alpha}{\beta - \alpha}, \quad c = \frac{\gamma}{\beta - \alpha} \quad (2)$$

with $-\alpha$ and $-\beta$ being the roots of the polynomial

$$\theta^2 + \theta(1 + n\gamma) + \gamma \quad (3)$$

whose discriminant is $\Delta = (1 + n\gamma)^2 - 4\gamma$, and therefore

$$\alpha = \frac{1}{2}\left(1 + n\gamma + \sqrt{\Delta}\right) \quad (4)$$

and

$$\beta = \frac{1}{2}\left(1 + n\gamma - \sqrt{\Delta}\right) \quad (5)$$

with $\gamma = \frac{M}{n-1} = \alpha\beta$.

In addition, the IICR for the case where the two genes are sampled in *different* demes ($IICR_d$), is given by the following:

$$IICR_d(t) = \frac{\frac{1}{\alpha}e^{-\alpha t} - \frac{1}{\beta}e^{-\beta t}}{e^{-\alpha t} - e^{-\beta t}} = \frac{\beta e^{-\alpha t} - \alpha e^{-\beta t}}{\gamma e^{-\alpha t} - \gamma e^{-\beta t}} \tag{6}$$

Mazet *et al.* (2016) noted that the $IICR_s$ and $IICR_d$ exhibited different trajectories. For the n-island model, the $IICR_s$ exhibits a history of monotonic decrease whereas the $IICR_d$ shows a history of expansion, hence stressing the importance of sampling on demographic inference as several authors have stressed (Wakeley, 1999; Beaumont, 2004; Städler *et al.*, 2009; Chikhi *et al.*, 2010; Heller *et al.*, 2013; Paz-Vinas *et al.*, 2013). Going further, and as noted above, the "demographic history" is sample-dependent.

For models for which no analytical results are known Mazet *et al.* (2016) noted that it is possible to estimate the IICR by simulating independent T_2 values and using them to estimate the IICR at various time points t_i, as follows:

$$\widehat{IICR(t_i)} = \frac{1 - \widehat{F_{T_2}(t_i)}}{\widehat{f_{T_2}(t_i)}} \tag{7}$$

where $\widehat{F_{T_2}(t_i)}$ is the estimated or empirical cumulative distribution function of T_2 and $\widehat{f_{T_2}(t_i)}$ is an estimated approximation of its density around t_i.

This computational approach can then be used to predict the IICR (and thus the PSMC plots) for any model of interest. To help identify among alternative scenarios those that can explain the genomic data and those that cannot, we provide below a flow-chart. This flow-chart is only provided to explain how the IICR could be used for model choice and model exclusion (figure S1). In a few words it explains how IICR plots should be produced and compared to observed PSMC plots. In the next section, we identify the models used in this study and for which we provided the IICR plots under several sampling schemes. In all cases data were simulated using the *ms* software (Hudson, 2002). For each scenario 10^6 independent T_2 values were simulated and the IICR computed and plotted with a python script available at https://github.com/willyrv/IICREstimator.

Before that and to complement the main manuscript we provide a figure explaining why the IICR is not (necessarily) an instantaneous effective size (N_e) under (some) structured models.

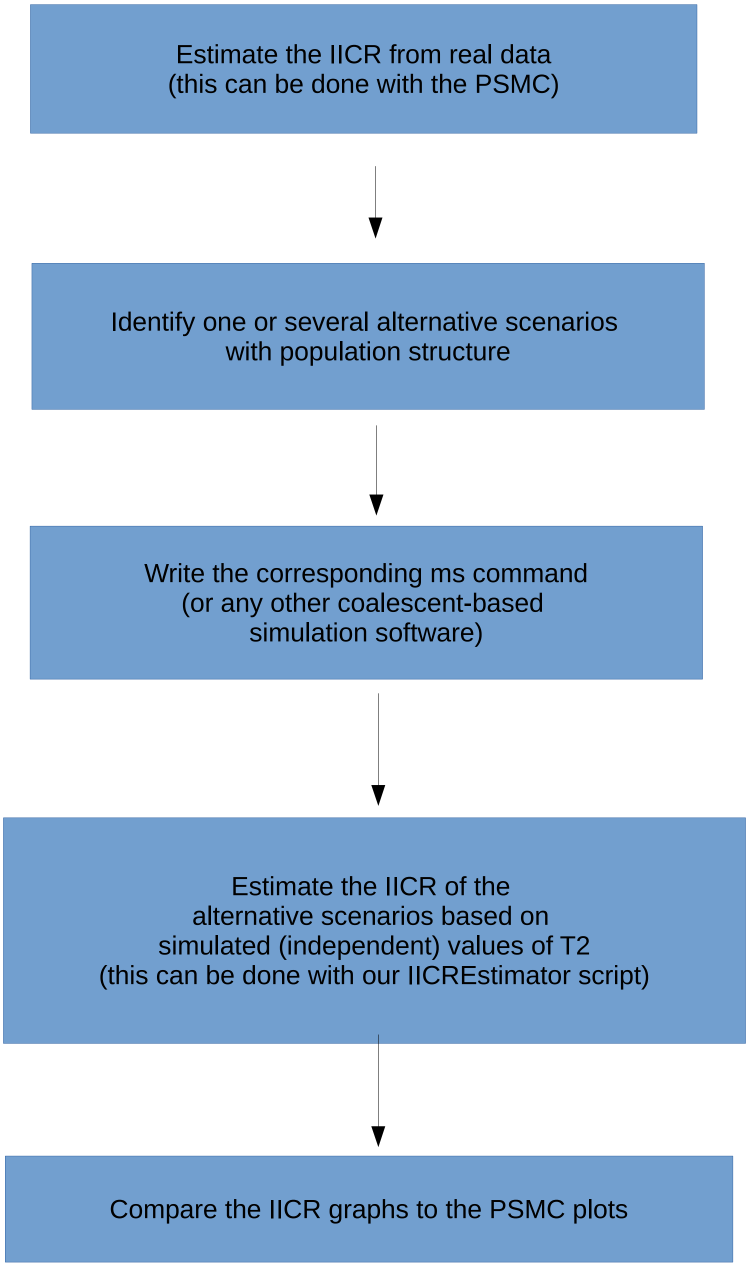

Figure S 1: Flow chart. This flow chart provides simplified guidelines on how to use our script to compare simulated IICR and observed PSMC plots. The script we used for estimating the IICR can be found at https://github.com/willyrv/IICREstimator.

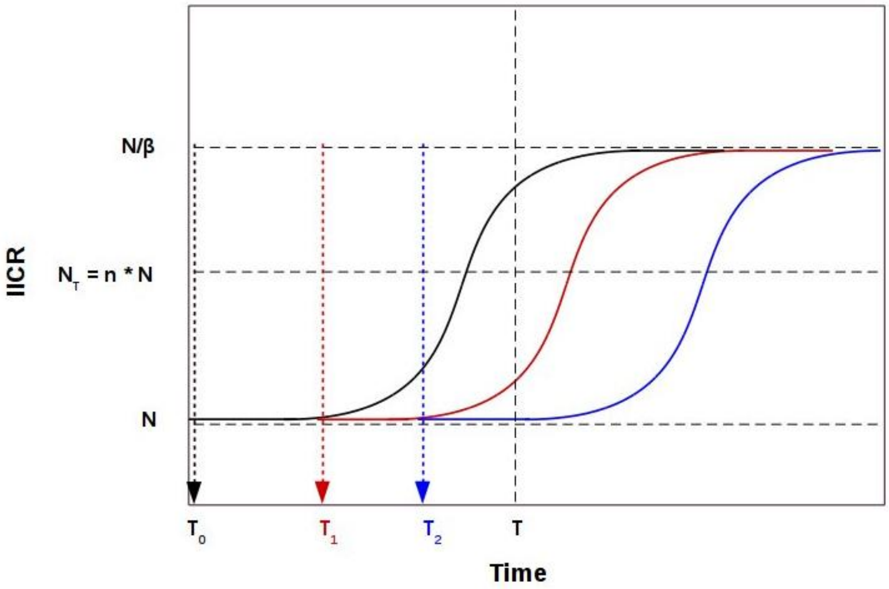

Figure S 2: IICR of the n-island model for three individuals sampled at different times. This figure shows a schematic representation of the IICR plots that characterize three diploid individuals sampled at three different times (T_0, T_1, and T_2) each in one deme of an n-island model. The IICR plots have an S-shape that necessarily starts at the time of sampling. The black line represents the IICR plot of an individual sampled in the present (T_0), whereas the red and blue lines represent IICR plots of two individuals sampled at different times in the past (T_1 and T_2, respectively). The dashed vertical line indicates some arbitrary time at which we ask what the instantaneous N_e is. If the notion of instantaneous N_e was meaningful here, the three IICR would point to the same value. This is not the case. The black line is close to N/β, the red line is larger than N, whereas the blue line indicates N.

2 IICR is not an instantaneous effective size

In the main manuscript we defend the notion that the IICR should not be seen and interpreted as an instantaneous N_e. The figure S2 is added to clarify the text in the main manuscript. The IICR avoids voluntarily the notion of effective size, and focuses on what is actually measured or estimated. We believe that this solves several problems related to its interpretation. The IICR incorporates the notion that coalescence rates can vary as a function of the sampling strategy. It is important because apparent changes in population size seen in the IICR are *constitutive* of the model and sample (location and timing). This is a crucial point for theoretical and practical reasons. The figure S2 shows how, by using three individuals sampled in a stationary structured model. Since we assume stationarity the IICR plots are simply shifted towards the past. Even if the shape of the IICR of an individual sampled today suggests that the instantaneous N_e was larger in the past (say at time T_2), a sample from that time point would not exhibit more diversity (since we assume a stationary model). That sample will have the same IICR except that it starts at that sample's time point (T_2, for instance) and is thus shifted towards the past. In the case of a real population size change, the ancient sample should exhibit a higher or lower diversity depending on its instantaneous N_e at that generation compared to the instantaneous N_e at time zero. Thus, in a structured population, the notion of instantaneous N_e as estimated from the IICR is potentially misleading (figure S2).

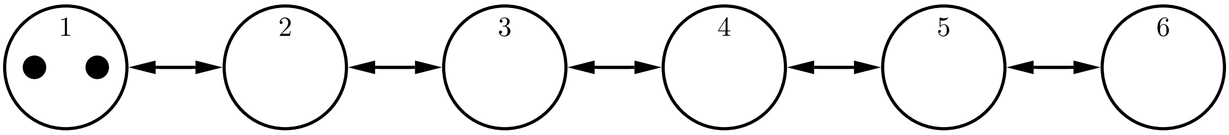

(a) $n = 6$

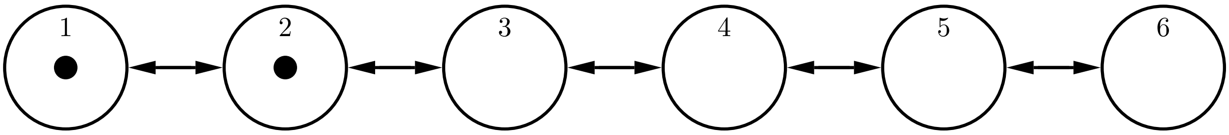

(b) $n = 6$

Figure S 3: 1D stepping stone model. Demes are numbered by row starting in the left side. In panel (a) the two genes were sampled in the same deme (deme 1), whereas in panel (b) they were sampled in different demes (deme 1 and 2). The same notation is used in all the simulations performed and sampling schemes used in this study.

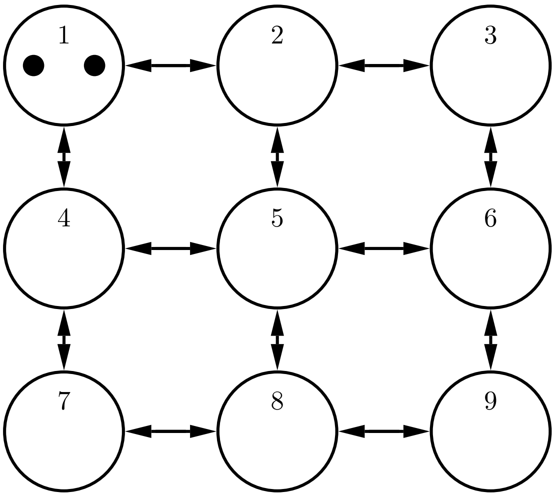

(a) $n = 9$

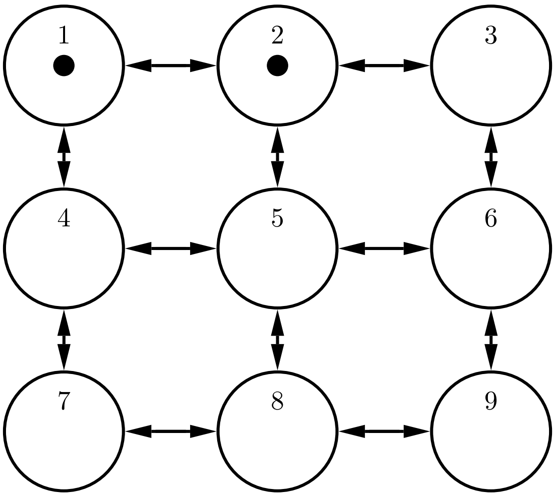

(b) $n = 9$

Figure S 4: 2D stepping stone model. Demes are numbered row by row starting with the top left corner deme In panel (a) the two genes were sampled in the same deme (deme 1), whereas in panel (b) they were sampled in different demes (demes 1 and 2).

3 Models

In this section we provide a description of all the models used in the manuscript.

3.1 stepping-stone models

The 2D stepping-stone model of Kimura and Weiss (1964) assumes that demes of size N haploid genomes are spatially organized in n_r rows and n_c columns, and that demes are only exchanging genes with their closest neighbours located in the same column and row (figure S4). The 1D stepping stone model (figure S3) assumes that all demes are arranged in a single row (*i.e.* $n_r = 1$).

3.2 n-island and asymmetrical island models

In the *n-islands* model (figure S5) a set of n islands (or demes) of constant size (N haploid genomes) are interconnected by symmetrical gene flow with a migration rate m, where $M = 2Nm$ is the number of immigrants (genes) in each island every generation. The asymmetrical gene flow models (figure S6a and S6b) assume that demes of size N haploid genomes are interconnected by gene flow with different migration rates.

The continent-island model (figure S7) assumes that demes with different sizes are interconnected by symmetrical gene flow with immigration rate m. Note that because m is an immigration rate a large population (here named the continent) is receiving more migrants (M_{12}) and sending less migrants (M_{21}) to a small population (here named the island).

3.3 Split models

Figure S8 shows population split models where one ancestral population of size N (figure S8a) or $2N$ (figure S8b) splits in two populations of size N at time T_{s1}. The first splitting model assumes an instantaneous doubling of the size during the splitting event whereas the second splitting model assumes that the total population size remains constant. Figure S8c shows a population split model where one ancestral population of size $3N$ splits in two populations of size N and $2N$ at time T_{s1}. The population of size $2N$ splits at time T_{s2} in two populations of size N. Figure S8d shows a population split model where one ancestral population of size N splits in two populations of size N at time T_{s1}. The second population splits at time T_{s2} in two populations of size N.

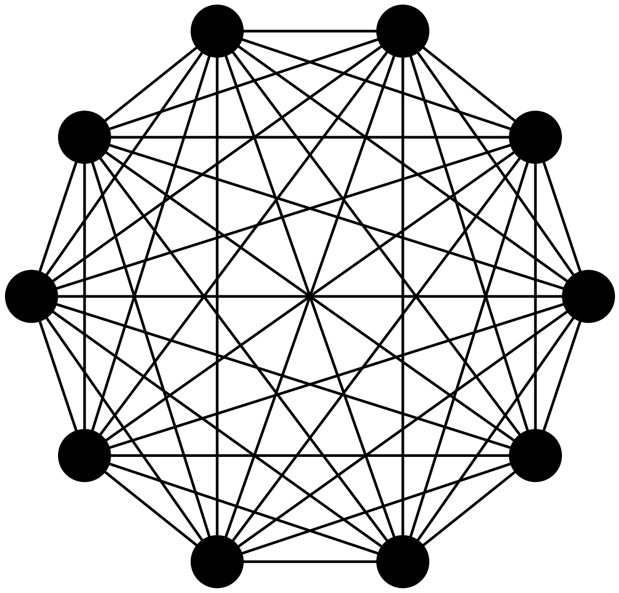

Figure S 5: *n-islands* model. Each circle represents a deme of size N and all demes are interconnected by symmetrical gene flow.

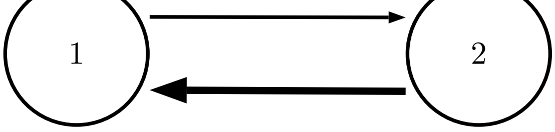

(a) Asymmetrical gene flow with two same-sized islands

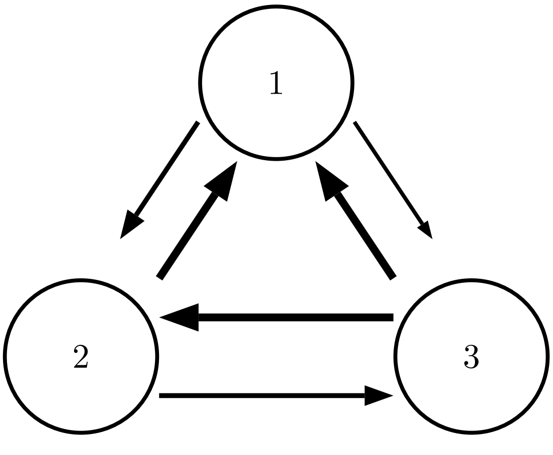

(b) Asymmetrical gene flow with three same-sized islands

Figure S 6: Asymmetrical gene flow with same-sized islands. The thickness of the arrows indicates higher or lower migration rates. In panel (a) deme 1 receive more migrants from deme 2, than deme 2 from deme 1. In panel (b) deme 1 receive more migrants than the other two demes. Deme 3 receive fewer migrants.

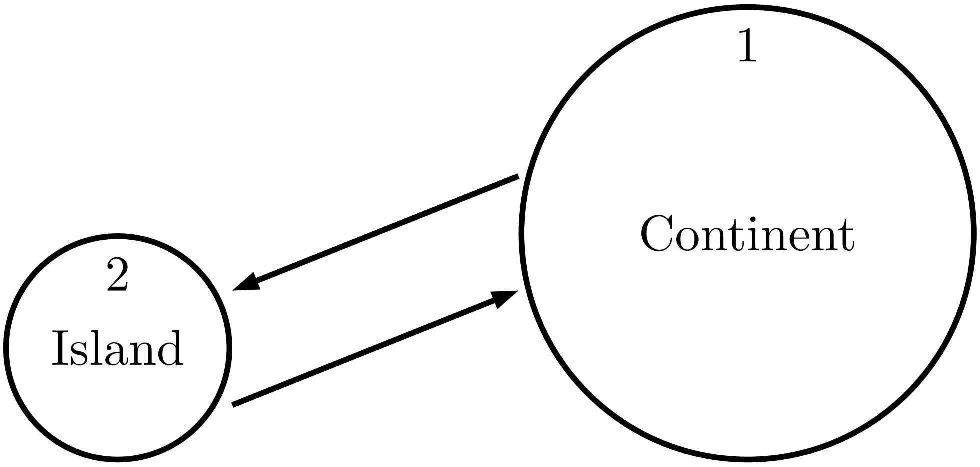

Figure S 7: Continent-island model. The two demes are interconnected by gene flow with an immigration rate m, but deme 1 (continent) is receiving more migrants than deme 2.

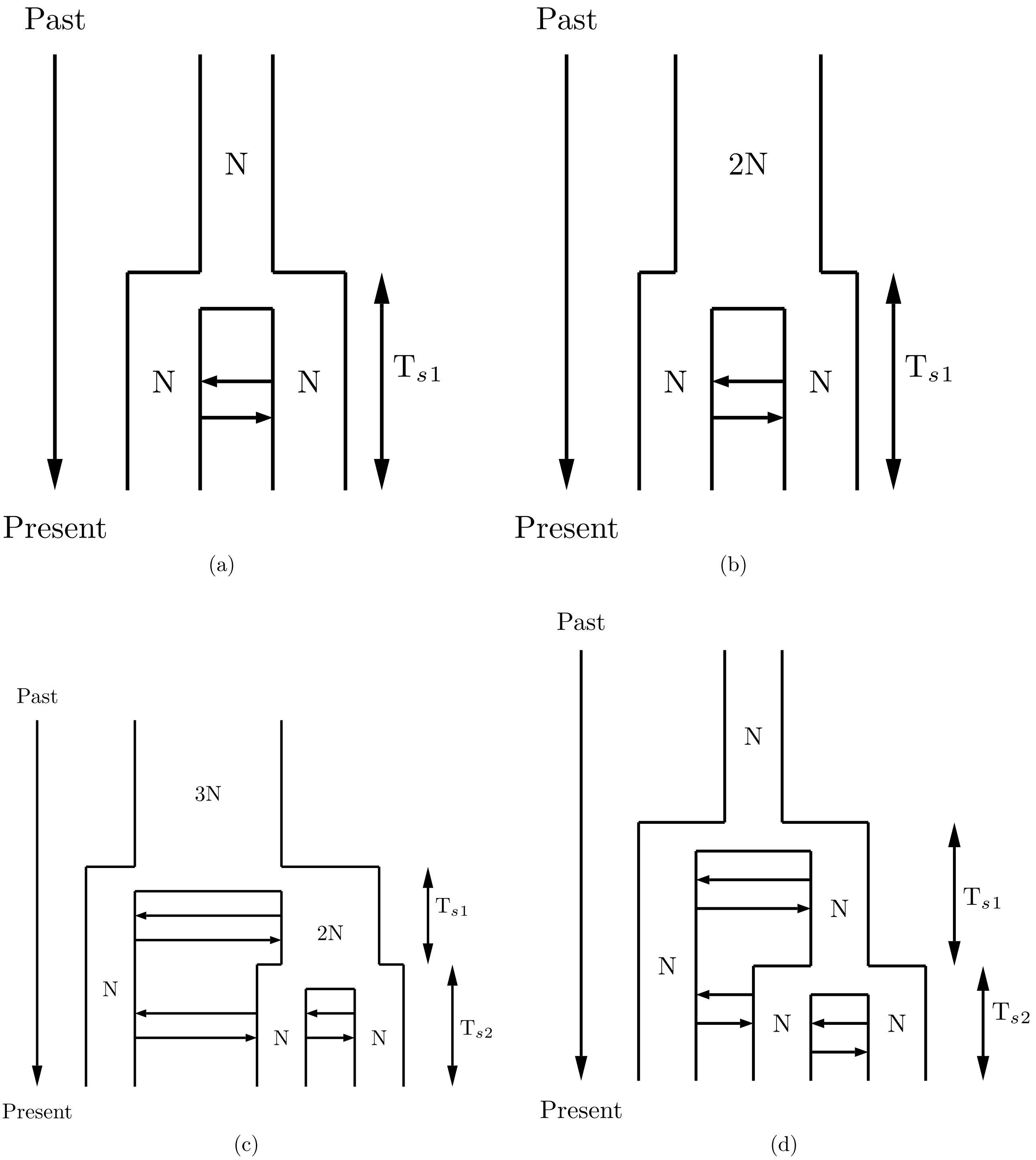

Figure S 8: Population split models. Panel (a) shows one ancestral population of size N splitting in two populations of size N at time T_{s1} assuming an instantaneous doubling of the size. Panel (b) shows one ancestral population of size $2N$ splitting in two populations of size N at time T_{s1} assuming a constant population size. Panel (c) shows one ancestral population of size $3N$ splitting in two populations of size N and $2N$ at time T_{s1}. The population of size $2N$ splits at time T_{s2} in two populations of size N. Panel (d) shows one ancestral population of size N splitting in two populations of size N at time T_{s1}. The second population splits at time T_{s2} in two populations of size N.

4 *ms* commands used for the figures of the main manuscript

Below we list the *ms* commands used for all the figures of the manuscript.

Figure 1 panel a:

---

ms 2 1000000 -T -L -I 6 2 0 0 0 0 0 1

ms 2 1000000 -T -L -I 6 2 0 0 0 0 0 -m 1 2 0.5 -m 2 1 0.5 -m 2 3 0.5 -m 3 2 0.5 -m 4 5 0.5 -m 5 4 0.5 -m 5 6 0.5 -m 6 5 0.5 -m 3 4 0.5 -m 4 3 0.5

ms 2 1000000 -T -L -I 6 0 2 0 0 0 0 -m 1 2 0.5 -m 2 1 0.5 -m 2 3 0.5 -m 3 2 0.5 -m 4 5 0.5 -m 5 4 0.5 -m 5 6 0.5 -m 6 5 0.5 -m 3 4 0.5 -m 4 3 0.5

ms 2 1000000 -T -L -I 6 0 0 2 0 0 0 -m 1 2 0.5 -m 2 1 0.5 -m 2 3 0.5 -m 3 2 0.5 -m 4 5 0.5 -m 5 4 0.5 -m 5 6 0.5 -m 6 5 0.5 -m 3 4 0.5 -m 4 3 0.5

Figure 1 panel b:

---

ms 2 1000000 -T -L -I 49 2 0 0 0 0 0 0 0 0 0 0 0 0 0 0 0 0 0 0 0 0 0 0 0 0 0 0 0 0 0 0 0 0 0 0 0 0 0 0 0 0 0 0 0 0 0 0 1

ms 2 1000000 -T -L -I 49 2 0 0 0 0 0 0 0 0 0 0 0 0 0 0 0 0 0 0 0 0 0 0 0 0 0 0 0 0 0 0 0 0 0 0 0 0 0 0 0 0 0 0 0 0 0 0 0 -m 1 8 0.25 -m 1 2 0.25 -m 2 9 0.25 -m 2 1 0.25 -m 2 3 0.25 -m 3 10 0.25 -m 3 2 0.25 -m 3 4 0.25 -m 4 11 0.25 -m 4 3 0.25 -m 4 5 0.25 -m 5 12 0.25 -m 5 4 0.25 -m 5 6 0.25 -m 6 13 0.25 -m 6 5 0.25 -m 6 7 0.25 -m 7 14 0.25 -m 7 6 0.25 -m 8 1 0.25 -m 8 15 0.25 -m 8 9 0.25 -m 9 2 0.25 -m 9 16 0.25 -m 9 8 0.25 -m 9 10 0.25 -m 10 3 0.25 -m 10 17 0.25 -m 10 9 0.25 -m 10 11 0.25 -m 11 4 0.25 -m 11 18 0.25 -m 11 10 0.25 -m 11 12 0.25 -m 12 5 0.25 -m 12 19 0.25 -m 12 11 0.25 -m 12 13 0.25 -m 13 6 0.25 -m 13 20 0.25 -m 13 12 0.25 -m 13 14 0.25 -m 14 7 0.25 -m 14 21 0.25 -m 14 13 0.25 -m 15 8 0.25 -m 15 22 0.25 -m 15 16 0.25 -m 16 9 0.25 -m 16 23 0.25 -m 16 15 0.25 -m 16 17 0.25 -m 17 10 0.25 -m 17 24 0.25 -m 17 16 0.25 -m 17 18 0.25 -m 18 11 0.25 -m 18 25 0.25 -m 18 17 0.25 -m 18 19 0.25 -m 19 12 0.25 -m 19 26 0.25 -m 19 18 0.25 -m 19 20 0.25 -m 20 13 0.25 -m 20 27 0.25 -m 20 19 0.25 -m 20 21 0.25 -m 21 14 0.25 -m 21 28 0.25 -m 21 20 0.25 -m 22 15 0.25 -m 22 29 0.25 -m 22 23 0.25 -m 23 16 0.25 -m 23 30 0.25 -m 23 22 0.25 -m 23 24 0.25 -m 24 17 0.25 -m 24 31 0.25 -m 24 23 0.25 -m 24 25 0.25 -m 25 18 0.25 -m 25 32 0.25 -m 25 24 0.25 -m 25 26 0.25 -m 26 19 0.25 -m 26 33 0.25 -m 26 25 0.25 -m 26 27 0.25 -m 27 20 0.25 -m 27 34 0.25 -m 27 26 0.25 -m 27 28 0.25 -m 28 21 0.25 -m 28 35 0.25 -m 28 27 0.25 -m 29 22 0.25 -m 29 36 0.25 -m 29 30 0.25 -m 30 23 0.25 -m 30 37 0.25 -m 30 29 0.25 -m 30 31 0.25 -m 31 24 0.25 -m 31 38 0.25 -m 31 30 0.25 -m 31 32 0.25 -m 32 25 0.25 -m 32 39 0.25 -m 32 31 0.25 -m 32 33 0.25 -m 33 26 0.25 -m 33 40 0.25 -m 33 32 0.25 -m 33 34 0.25 -m 34 27 0.25 -m 34 41 0.25 -m 34 33 0.25 -m 34 35 0.25 -m 35 28 0.25 -m 35 42 0.25 -m 35 34 0.25 -m 36 29 0.25 -m 36 43 0.25 -m 36 37 0.25 -m 37 30 0.25 -m 37 44 0.25 -m 37 36 0.25 -m 37 38 0.25 -m 38 31 0.25 -m 38 45 0.25 -m 38 37 0.25 -m 38 39 0.25 -m 39 32 0.25 -m 39 46 0.25 -m 39 38 0.25 -m 39 40 0.25 -m 40 33 0.25 -m 40 47 0.25 -m 40 39 0.25 -m 40 41 0.25 -m 41 34 0.25 -m 41 48 0.25 -m 41 40 0.25 -m 41 42 0.25 -m 42 35 0.25 -m 42 49 0.25 -m 42 41 0.25 -m 43 36 0.25 -m 43 44 0.25 -m 44 37 0.25 -m 44 43 0.25 -m 44 45 0.25 -m 45 38 0.25 -m 45 44 0.25 -m 45 46 0.25 -m 46 39 0.25 -m 46 45 0.25 -m 46 47 0.25 -m 47 40 0.25 -m 47 46 0.25 -m 47 48 0.25 -m 48 41 0.25 -m 48 47 0.25 -m 48 49 0.25 -m 49 42 0.25 -m 49 48 0.25

ms 2 1000000 -T -L -I 49 0 0 0 2 0 0 0 0 0 0 0 0 0 0 0 0 0 0 0 0 0 0 0 0 0 0 0 0 0 0 0 0 0 0 0 0 0 0 0 0 0 0 0 0 0 0 0 0 -m 1 8 0.25 -m 1 2 0.25 -m 2 9 0.25 -m 2 1 0.25 -m 2 3 0.25 -m 3 10 0.25 -m 3 2 0.25 -m 3 4 0.25 -m 4 11 0.25 -m 4 3 0.25 -m 4 5 0.25 -m 5 12 0.25 -m 5 4 0.25 -m 5 6 0.25 -m 6 13 0.25 -m 6 5 0.25 -m 6 7 0.25 -m 7 14 0.25 -m 7 6 0.25 -m 8 1 0.25 -m 8 15 0.25 -m 8 9 0.25 -m 9 2 0.25 -m 9 16 0.25 -m 9 8 0.25 -m 9 10 0.25 -m 10 3 0.25 -m 10 17 0.25 -m 10 9 0.25 -m 10 11 0.25 -m 11 4 0.25 -m 11 18 0.25 -m 11 10 0.25 -m 11 12 0.25 -m 12 5 0.25 -m 12 19 0.25 -m 12 11 0.25 -m 12 13 0.25 -m 13 6 0.25 -m 13 20 0.25 -m 13 12 0.25 -m 13 14 0.25 -m 14 7 0.25 -m 14 21 0.25 -m 14 13 0.25 -m 15 8 0.25 -m 15 22 0.25 -m 15 16 0.25 -m 16 9 0.25 -m 16 23 0.25 -m 16 15 0.25 -m 16 17 0.25 -m 17 10 0.25 -m 17 24 0.25 -m 17 16 0.25 -m 17 18 0.25 -m 18 11 0.25 -m 18 25 0.25 -m 18 17 0.25 -m 18 19 0.25 -m 19 12 0.25 -m 19 26 0.25 -m 19 18 0.25 -m 19 20 0.25 -m 20 13 0.25 -m 20 27 0.25 -m 20 19 0.25 -m 20 21 0.25 -m 21 14 0.25 -m 21 28 0.25 -m 21 20 0.25 -m 22 15 0.25 -m 22 29 0.25 -m 22 23 0.25 -m 23 16 0.25 -m 23 30 0.25 -m 23 22 0.25 -m 23 24 0.25 -m 24 17 0.25 -m 24 31 0.25 -m 24 23 0.25 -m 24 25 0.25 -m 25 18 0.25 -m 25 32 0.25 -m 25 24 0.25 -m 25 26 0.25 -m 26 19 0.25 -m 26 33 0.25 -m 26 25 0.25 -m 26 27 0.25 -m 27 20 0.25 -m 27 34 0.25 -m 27 26 0.25 -m 27 28 0.25 -m 28 21 0.25 -m 28 35 0.25 -m 28 27 0.25 -m 29 22 0.25 -m 29 36 0.25 -m 29 30 0.25 -m 30 23 0.25 -m 30 37 0.25 -m 30 29 0.25 -m 30 31 0.25 -m 31 24 0.25 -m 31 38 0.25 -m 31 30 0.25 -m 31 32 0.25 -m 32 25 0.25 -m 32 39 0.25 -m 32 31 0.25 -m 32 33 0.25 -m 33 26 0.25 -m 33 40 0.25 -m 33 32 0.25 -m 33 34 0.25 -m 34 27 0.25 -m 34 41 0.25 -m 34 33 0.25 -m 34 35 0.25 -m 35 28 0.25 -m 35 42 0.25 -m 35 34 0.25 -m 36 29 0.25 -m 36 43 0.25 -m 36 37 0.25 -m 37 30 0.25 -m 37 44 0.25 -m 37 36 0.25 -m 37 38 0.25 -m 38 31 0.25 -m 38 45 0.25 -m 38 37 0.25 -m 38 39 0.25 -m 39 32 0.25 -m 39 46 0.25 -m 39 38 0.25 -m 39 40 0.25 -m 40 33 0.25 -m 40 47 0.25 -m 40 39 0.25 -m 40 41 0.25 -m 41 34 0.25 -m 41 48 0.25 -m 41 40 0.25 -m 41 42 0.25 -m 42 35 0.25 -m 42 49 0.25 -m 42 41 0.25 -m 43 36 0.25 -m 43 44 0.25 -m 44 37 0.25 -m 44 43 0.25 -m 44 45 0.25 -m 45 38 0.25 -m 45 44 0.25 -m 45 46 0.25 -m 46 39 0.25 -m 46 45 0.25 -m 46 47 0.25 -m 47 40 0.25 -m 47 46 0.25 -m 47 48 0.25 -m 48 41 0.25 -m 48 47 0.25 -m 48 49 0.25 -m 49 42 0.25 -m 49 48 0.25

ms 2 1000000 -T -L -I 49 0 0 0 0 0 0 0 0 0 0 0 0 0 0 0 0 0 0 0 0 0 0 0 0 2 0 0 0 0 0 0 0 0 0 0 0 0 0 0 0 0 0 0 0 0 0 0 0 -m 1 8 0.25 -m 1 2 0.25 -m 2 9 0.25 -m 2 1 0.25 -m 2 3 0.25 -m 3 10 0.25 -m 3 2 0.25 -m 3 4 0.25 -m 4 11 0.25 -m 4 3 0.25 -m 4 5 0.25 -m 5 12 0.25 -m 5 4 0.25 -m 5 6 0.25 -m 6 13 0.25 -m 6 5 0.25 -m 6 7 0.25 -m 7 14 0.25 -m 7 6 0.25 -m 8 1 0.25 -m 8 15 0.25 -m 8 9 0.25 -m 9 2 0.25 -m 9 16 0.25 -m 9 8 0.25 -m 9 10 0.25 -m 10 3 0.25 -m 10 17 0.25 -m 10 9 0.25 -m 10 11 0.25 -m 11 4 0.25 -m 11 18 0.25 -m 11 10 0.25 -m 11 12 0.25 -m 12 5 0.25 -m 12 19 0.25 -m 12 11 0.25 -m 12 13 0.25 -m 13 6 0.25 -m 13 20 0.25 -m 13 12 0.25 -m 13 14 0.25 -m 14 7 0.25 -m 14 21 0.25 -m 14 13 0.25 -m 15 8 0.25 -m 15 22 0.25 -m 15 16 0.25 -m 16 9 0.25 -m 16 23 0.25 -m 16 15 0.25 -m 16 17 0.25 -m 17 10 0.25 -m 17 24 0.25 -m 17 16 0.25 -m 17 18 0.25 -m 18 11 0.25 -m 18 25 0.25 -m 18 17 0.25 -m 18 19 0.25 -m 19 12 0.25 -m 19 26 0.25 -m 19 18 0.25 -m 19 20 0.25 -m 20 13 0.25 -m 20 27 0.25 -m 20 19 0.25 -m 20 21 0.25 -m 21 14 0.25 -m 21 28 0.25 -m 21 20 0.25 -m 22 15 0.25 -m 22 29 0.25 -m 22 23 0.25 -m 23 16 0.25 -m 23 30 0.25 -m 23 22 0.25 -m 23 24 0.25 -m 24 17 0.25 -m 24 31 0.25 -m 24 23 0.25 -m 24 25 0.25 -m 25 18 0.25 -m 25 32 0.25 -m 25 24 0.25 -m 25 26 0.25 -m 26 19 0.25 -m 26 33 0.25 -m 26 25 0.25 -m 26 27 0.25 -m 27 20 0.25 -m 27 34 0.25 -m 27 26 0.25 -m 27 28 0.25 -m 28 21 0.25 -m 28 35 0.25 -m 28 27 0.25 -m 29 22 0.25 -m 29 36 0.25 -m 29 30 0.25 -m 30 23 0.25 -m 30 37 0.25 -m 30 29 0.25 -m 30 31 0.25 -m 31 24 0.25 -m 31 38 0.25 -m 31 30 0.25 -m 31 32 0.25 -m 32 25 0.25 -m 32 39 0.25 -m 32 31 0.25 -m 32 33 0.25 -m 33 26 0.25 -m 33 40 0.25 -m 33 32 0.25 -m 33 34 0.25 -m 34 27 0.25 -m 34 41 0.25 -m 34 33 0.25 -m 34 35 0.25 -m 35 28 0.25 -m 35 42 0.25 -m 35 34 0.25 -m 36 29 0.25 -m 36 43 0.25 -m 36 37 0.25 -m 37 30 0.25 -m 37 44 0.25 -m 37 36 0.25 -m 37 38 0.25 -m 38 31 0.25 -m 38 45 0.25 -m 38 37 0.25 -m 38 39 0.25 -m 39 32 0.25 -m 39 46 0.25 -m 39 38 0.25 -m 39 40 0.25 -m 40 33 0.25 -m 40 47 0.25 -m 40 39 0.25 -m 40 41 0.25 -m 41 34 0.25 -m 41 48 0.25 -m 41 40 0.25 -m 41 42 0.25 -m 42 35 0.25 -m 42 49 0.25 -m 42 41 0.25 -m 43 36 0.25 -m 43 44 0.25 -m 44 37 0.25 -m 44 43 0.25 -m 44 45 0.25 -m 45 38 0.25 -m 45 44 0.25 -m 45 46 0.25 -m 46 39 0.25

-m 46 45 0.25 -m 46 47 0.25 -m 47 40 0.25 -m 47 46 0.25 -m 47 48 0.25 -m 48 41 0.25 -m 48 47 0.25 -m 48 49 0.25 -m 49 42 0.25 -m 49 48 0.25

Figure 1 panel c:

---

ms 2 1000000 -T -L -I 6 1 1 0 0 0 0 1

ms 2 1000000 -T -L -I 6 1 1 0 0 0 0 -m 1 2 0.5 -m 2 1 0.5 -m 2 3 0.5 -m 3 2 0.5 -m 4 5 0.5 -m 5 4 0.5 -m 5 6 0.5 -m 6 5 0.5 -m 3 4 0.5 -m 4 3 0.5

ms 2 1000000 -T -L -I 6 1 0 1 0 0 0 -m 1 2 0.5 -m 2 1 0.5 -m 2 3 0.5 -m 3 2 0.5 -m 4 5 0.5 -m 5 4 0.5 -m 5 6 0.5 -m 6 5 0.5 -m 3 4 0.5 -m 4 3 0.5

ms 2 1000000 -T -L -I 6 1 0 0 1 0 0 -m 1 2 0.5 -m 2 1 0.5 -m 2 3 0.5 -m 3 2 0.5 -m 4 5 0.5 -m 5 4 0.5 -m 5 6 0.5 -m 6 5 0.5 -m 3 4 0.5 -m 4 3 0.5

ms 2 1000000 -T -L -I 6 0 0 1 1 0 0 -m 1 2 0.5 -m 2 1 0.5 -m 2 3 0.5 -m 3 2 0.5 -m 4 5 0.5 -m 5 4 0.5 -m 5 6 0.5 -m 6 5 0.5 -m 3 4 0.5 -m 4 3 0.5

Figure 1 panel d:

---

ms 2 1000000 -T -L -I 49 1 1 0 0 0 0 0 0 0 0 0 0 0 0 0 0 0 0 0 0 0 0 0 0 0 0 0 0 0 0 0 0 0 0 0 0 0 0 0 0 0 0 0 0 0 0 0 0 1

ms 2 1000000 -T -L -I 49 1 1 0 0 0 0 0 0 0 0 0 0 0 0 0 0 0 0 0 0 0 0 0 0 0 0 0 0 0 0 0 0 0 0 0 0 0 0 0 0 0 0 0 0 0 0 0 0 -m 1 8 0.25 -m 1 2 0.25 -m 2 9 0.25 -m 2 1 0.25 -m 2 3 0.25 -m 3 10 0.25 -m 3 2 0.25 -m 3 4 0.25 -m 4 11 0.25 -m 4 3 0.25 -m 4 5 0.25 -m 5 12 0.25 -m 5 4 0.25 -m 5 6 0.25 -m 6 13 0.25 -m 6 5 0.25 -m 6 7 0.25 -m 7 14 0.25 -m 7 6 0.25 -m 8 1 0.25 -m 8 15 0.25 -m 8 9 0.25 -m 9 2 0.25 -m 9 16 0.25 -m 9 8 0.25 -m 9 10 0.25 -m 10 3 0.25 -m 10 17 0.25 -m 10 9 0.25 -m 10 11 0.25 -m 11 4 0.25 -m 11 18 0.25 -m 11 10 0.25 -m 11 12 0.25 -m 12 5 0.25 -m 12 19 0.25 -m 12 11 0.25 -m 12 13 0.25 -m 13 6 0.25 -m 13 20 0.25 -m 13 12 0.25 -m 13 14 0.25 -m 14 7 0.25 -m 14 21 0.25 -m 14 13 0.25 -m 15 8 0.25 -m 15 22 0.25 -m 15 16 0.25 -m 16 9 0.25 -m 16 23 0.25 -m 16 15 0.25 -m 16 17 0.25 -m 17 10 0.25 -m 17 24 0.25 -m 17 16 0.25 -m 17 18 0.25 -m 18 11 0.25 -m 18 25 0.25 -m 18 17 0.25 -m 18 19 0.25 -m 19 12 0.25 -m 19 26 0.25 -m 19 18 0.25 -m 19 20 0.25 -m 20 13 0.25 -m 20 27 0.25 -m 20 19 0.25 -m 20 21 0.25 -m 21 14 0.25 -m 21 28 0.25 -m 21 20 0.25 -m 22 15 0.25 -m 22 29 0.25 -m 22 23 0.25 -m 23 16 0.25 -m 23 30 0.25 -m 23 22 0.25 -m 23 24 0.25 -m 24 17 0.25 -m 24 31 0.25 -m 24 23 0.25 -m 24 25 0.25 -m 25 18 0.25 -m 25 32 0.25 -m 25 24 0.25 -m 25 26 0.25 -m 26 19 0.25 -m 26 33 0.25 -m 26 25 0.25 -m 26 27 0.25 -m 27 20 0.25 -m 27 34 0.25 -m 27 26 0.25 -m 27 28 0.25 -m 28 21 0.25 -m 28 35 0.25 -m 28 27 0.25 -m 29 22 0.25 -m 29 36 0.25 -m 29 30 0.25 -m 30 23 0.25 -m 30 37 0.25 -m 30 29 0.25 -m 30 31 0.25 -m 31 24 0.25 -m 31 38 0.25 -m 31 30 0.25 -m 31 32 0.25 -m 32 25 0.25 -m 32 39 0.25 -m 32 31 0.25 -m 32 33 0.25 -m 33 26 0.25 -m 33 40 0.25 -m 33 32 0.25 -m 33 34 0.25 -m 34 27 0.25 -m 34 41 0.25 -m 34 33 0.25 -m 34 35 0.25 -m 35 28 0.25 -m 35 42 0.25 -m 35 34 0.25 -m 36 29 0.25 -m 36 43 0.25 -m 36 37 0.25 -m 37 30 0.25 -m 37 44 0.25 -m 37 36 0.25 -m 37 38 0.25 -m 38 31 0.25 -m 38 45 0.25 -m 38 37 0.25 -m 38 39 0.25 -m 39 32 0.25 -m 39 46 0.25 -m 39 38 0.25 -m 39 40 0.25 -m 40 33 0.25 -m 40 47 0.25 -m 40 39 0.25 -m 40 41 0.25 -m 41 34 0.25 -m 41 48 0.25 -m 41

40 0.25 -m 41 42 0.25 -m 42 35 0.25 -m 42 49 0.25 -m 42 41 0.25 -m 43 36 0.25 -m 43 44 0.25 -m 44 37 0.25 -m 44 43 0.25 -m 44 45 0.25 -m 45 38 0.25 -m 45 44 0.25 -m 45 46 0.25 -m 46 39 0.25 -m 46 45 0.25 -m 46 47 0.25 -m 47 40 0.25 -m 47 46 0.25 -m 47 48 0.25 -m 48 41 0.25 -m 48 47 0.25 -m 48 49 0.25 -m 49 42 0.25 -m 49 48 0.25

ms 2 1000000 -T -L -I 49 1 0 1 0 0 0 0 0 0 0 0 0 0 0 0 0 0 0 0 0 0 0 0 0 0 0 0 0 0 0 0 0 0 0 0 0 0 0 0 0 0 0 0 0 0 0 0 0 0 -m 1 8 0.25 -m 1 2 0.25 -m 2 9 0.25 -m 2 1 0.25 -m 2 3 0.25 -m 3 10 0.25 -m 3 2 0.25 -m 3 4 0.25 -m 4 11 0.25 -m 4 3 0.25 -m 4 5 0.25 -m 5 12 0.25 -m 5 4 0.25 -m 5 6 0.25 -m 6 13 0.25 -m 6 5 0.25 -m 6 7 0.25 -m 7 14 0.25 -m 7 6 0.25 -m 8 1 0.25 -m 8 15 0.25 -m 8 9 0.25 -m 9 2 0.25 -m 9 16 0.25 -m 9 8 0.25 -m 9 10 0.25 -m 10 3 0.25 -m 10 17 0.25 -m 10 9 0.25 -m 10 11 0.25 -m 11 4 0.25 -m 11 18 0.25 -m 11 10 0.25 -m 11 12 0.25 -m 12 5 0.25 -m 12 19 0.25 -m 12 11 0.25 -m 12 13 0.25 -m 13 6 0.25 -m 13 20 0.25 -m 13 12 0.25 -m 13 14 0.25 -m 14 7 0.25 -m 14 21 0.25 -m 14 13 0.25 -m 15 8 0.25 -m 15 22 0.25 -m 15 16 0.25 -m 16 9 0.25 -m 16 23 0.25 -m 16 15 0.25 -m 16 17 0.25 -m 17 10 0.25 -m 17 24 0.25 -m 17 16 0.25 -m 17 18 0.25 -m 18 11 0.25 -m 18 25 0.25 -m 18 17 0.25 -m 18 19 0.25 -m 19 12 0.25 -m 19 26 0.25 -m 19 18 0.25 -m 19 20 0.25 -m 20 13 0.25 -m 20 27 0.25 -m 20 19 0.25 -m 20 21 0.25 -m 21 14 0.25 -m 21 28 0.25 -m 21 20 0.25 -m 22 15 0.25 -m 22 29 0.25 -m 22 23 0.25 -m 23 16 0.25 -m 23 30 0.25 -m 23 22 0.25 -m 23 24 0.25 -m 24 17 0.25 -m 24 31 0.25 -m 24 23 0.25 -m 24 25 0.25 -m 25 18 0.25 -m 25 32 0.25 -m 25 24 0.25 -m 25 26 0.25 -m 26 19 0.25 -m 26 33 0.25 -m 26 25 0.25 -m 26 27 0.25 -m 27 20 0.25 -m 27 34 0.25 -m 27 26 0.25 -m 27 28 0.25 -m 28 21 0.25 -m 28 35 0.25 -m 28 27 0.25 -m 29 22 0.25 -m 29 36 0.25 -m 29 30 0.25 -m 30 23 0.25 -m 30 37 0.25 -m 30 29 0.25 -m 30 31 0.25 -m 31 24 0.25 -m 31 38 0.25 -m 31 30 0.25 -m 31 32 0.25 -m 32 25 0.25 -m 32 39 0.25 -m 32 31 0.25 -m 32 33 0.25 -m 33 26 0.25 -m 33 40 0.25 -m 33 32 0.25 -m 33 34 0.25 -m 34 27 0.25 -m 34 41 0.25 -m 34 33 0.25 -m 34 35 0.25 -m 35 28 0.25 -m 35 42 0.25 -m 35 34 0.25 -m 36 29 0.25 -m 36 43 0.25 -m 36 37 0.25 -m 37 30 0.25 -m 37 44 0.25 -m 37 36 0.25 -m 37 38 0.25 -m 38 31 0.25 -m 38 45 0.25 -m 38 37 0.25 -m 38 39 0.25 -m 39 32 0.25 -m 39 46 0.25 -m 39 38 0.25 -m 39 40 0.25 -m 40 33 0.25 -m 40 47 0.25 -m 40 39 0.25 -m 40 41 0.25 -m 41 34 0.25 -m 41 48 0.25 -m 41 40 0.25 -m 41 42 0.25 -m 42 35 0.25 -m 42 49 0.25 -m 42 41 0.25 -m 43 36 0.25 -m 43 44 0.25 -m 44 37 0.25 -m 44 43 0.25 -m 44 45 0.25 -m 45 38 0.25 -m 45 44 0.25 -m 45 46 0.25 -m 46 39 0.25 -m 46 45 0.25 -m 46 47 0.25 -m 47 40 0.25 -m 47 46 0.25 -m 47 48 0.25 -m 48 41 0.25 -m 48 47 0.25 -m 48 49 0.25 -m 49 42 0.25 -m 49 48 0.25

ms 2 1000000 -T -L -I 49 1 0 0 1 0 0 0 0 0 0 0 0 0 0 0 0 0 0 0 0 0 0 0 0 0 0 0 0 0 0 0 0 0 0 0 0 0 0 0 0 0 0 0 0 0 0 0 0 0 -m 1 8 0.25 -m 1 2 0.25 -m 2 9 0.25 -m 2 1 0.25 -m 2 3 0.25 -m 3 10 0.25 -m 3 2 0.25 -m 3 4 0.25 -m 4 11 0.25 -m 4 3 0.25 -m 4 5 0.25 -m 5 12 0.25 -m 5 4 0.25 -m 5 6 0.25 -m 6 13 0.25 -m 6 5 0.25 -m 6 7 0.25 -m 7 14 0.25 -m 7 6 0.25 -m 8 1 0.25 -m 8 15 0.25 -m 8 9 0.25 -m 9 2 0.25 -m 9 16 0.25 -m 9 8 0.25 -m 9 10 0.25 -m 10 3 0.25 -m 10 17 0.25 -m 10 9 0.25 -m 10 11 0.25 -m 11 4 0.25 -m 11 18 0.25 -m 11 10 0.25 -m 11 12 0.25 -m 12 5 0.25 -m 12 19 0.25 -m 12 11 0.25 -m 12 13 0.25 -m 13 6 0.25 -m 13 20 0.25 -m 13 12 0.25 -m 13 14 0.25 -m 14 7 0.25 -m 14 21 0.25 -m 14 13 0.25 -m 15 8 0.25 -m 15 22 0.25 -m 15 16 0.25 -m 16 9 0.25 -m 16 23 0.25 -m 16 15 0.25 -m 16 17 0.25 -m 17 10 0.25 -m 17 24 0.25 -m 17 16 0.25 -m 17 18 0.25 -m 18 11 0.25 -m 18 25 0.25 -m 18 17 0.25 -m 18 19 0.25 -m 19 12 0.25 -m 19 26 0.25 -m 19 18 0.25 -m 19 20 0.25 -m 20 13 0.25 -m 20 27 0.25 -m 20 19 0.25 -m 20 21 0.25 -m 21 14 0.25 -m 21 28 0.25 -m 21 20 0.25 -m 22 15 0.25 -m 22 29 0.25 -m 22 23 0.25 -m 23 16 0.25 -m 23 30 0.25 -m 23 22 0.25 -m 23 24 0.25 -m 24 17 0.25 -m 24 31 0.25 -m 24 23 0.25 -m 24 25 0.25 -m 25 18 0.25 -m 25 32 0.25 -m 25 24 0.25 -m 25 26 0.25 -m 26 19 0.25 -m 26 33 0.25 -m 26 25 0.25 -m 26 27 0.25 -m 27 20 0.25 -m 27 34 0.25 -m 27 26 0.25 -m 27 28 0.25 -m 28 21 0.25 -m 28 35 0.25 -m 28 27 0.25 -m 29 22 0.25 -m 29 36 0.25 -m 29 30 0.25 -m 30 23 0.25 -m 30 37 0.25 -m 30 29 0.25 -m 30 31 0.25 -m 31 24 0.25 -m 31 38 0.25 -m 31 30 0.25 -m 31 32 0.25 -m 32 25 0.25 -m 32 39 0.25 -m 32 31 0.25 -m 32 33 0.25 -m 33 26 0.25 -m 33 40 0.25 -m 33 32 0.25 -m 33 34 0.25 -m 34 27 0.25 -m 34 41 0.25 -m 34 33 0.25 -m 34 35 0.25 -m 35 28 0.25 -m 35 42 0.25 -m 35 34 0.25 -m 36 29 0.25 -m 36 43 0.25 -m 36 37 0.25 -m 37 30 0.25 -m 37 44 0.25 -m 37 36 0.25 -m 37 38 0.25 -m 38 31 0.25

-m 38 45 0.25 -m 38 37 0.25 -m 38 39 0.25 -m 39 32 0.25 -m 39 46 0.25 -m 39 38 0.25 -m 39 40 0.25 -m 40 33 0.25 -m 40 47 0.25 -m 40 39 0.25 -m 40 41 0.25 -m 41 34 0.25 -m 41 48 0.25 -m 41 40 0.25 -m 41 42 0.25 -m 42 35 0.25 -m 42 49 0.25 -m 42 41 0.25 -m 43 36 0.25 -m 43 44 0.25 -m 44 37 0.25 -m 44 43 0.25 -m 44 45 0.25 -m 45 38 0.25 -m 45 44 0.25 -m 45 46 0.25 -m 46 39 0.25 -m 46 45 0.25 -m 46 47 0.25 -m 47 40 0.25 -m 47 46 0.25 -m 47 48 0.25 -m 48 41 0.25 -m 48 47 0.25 -m 48 49 0.25 -m 49 42 0.25 -m 49 48 0.25

ms 2 1000000 -T -L -I 49 1 0 0 0 0 0 0 0 0 0 0 0 0 0 0 0 0 0 0 0 0 0 0 0 1 0 0 0 0 0 0 0 0 0 0 0 0 0 0 0 0 0 0 0 0 0 0 0 0 -m 1 8 0.25 -m 1 2 0.25 -m 2 9 0.25 -m 2 1 0.25 -m 2 3 0.25 -m 3 10 0.25 -m 3 2 0.25 -m 3 4 0.25 -m 4 11 0.25 -m 4 3 0.25 -m 4 5 0.25 -m 5 12 0.25 -m 5 4 0.25 -m 5 6 0.25 -m 6 13 0.25 -m 6 5 0.25 -m 6 7 0.25 -m 7 14 0.25 -m 7 6 0.25 -m 8 1 0.25 -m 8 15 0.25 -m 8 9 0.25 -m 9 2 0.25 -m 9 16 0.25 -m 9 8 0.25 -m 9 10 0.25 -m 10 3 0.25 -m 10 17 0.25 -m 10 9 0.25 -m 10 11 0.25 -m 11 4 0.25 -m 11 18 0.25 -m 11 10 0.25 -m 11 12 0.25 -m 12 5 0.25 -m 12 19 0.25 -m 12 11 0.25 -m 12 13 0.25 -m 13 6 0.25 -m 13 20 0.25 -m 13 12 0.25 -m 13 14 0.25 -m 14 7 0.25 -m 14 21 0.25 -m 14 13 0.25 -m 15 8 0.25 -m 15 22 0.25 -m 15 16 0.25 -m 16 9 0.25 -m 16 23 0.25 -m 16 15 0.25 -m 16 17 0.25 -m 17 10 0.25 -m 17 24 0.25 -m 17 16 0.25 -m 17 18 0.25 -m 18 11 0.25 -m 18 25 0.25 -m 18 17 0.25 -m 18 19 0.25 -m 19 12 0.25 -m 19 26 0.25 -m 19 18 0.25 -m 19 20 0.25 -m 20 13 0.25 -m 20 27 0.25 -m 20 19 0.25 -m 20 21 0.25 -m 21 14 0.25 -m 21 28 0.25 -m 21 20 0.25 -m 22 15 0.25 -m 22 29 0.25 -m 22 23 0.25 -m 23 16 0.25 -m 23 30 0.25 -m 23 22 0.25 -m 23 24 0.25 -m 24 17 0.25 -m 24 31 0.25 -m 24 23 0.25 -m 24 25 0.25 -m 25 18 0.25 -m 25 32 0.25 -m 25 24 0.25 -m 25 26 0.25 -m 26 19 0.25 -m 26 33 0.25 -m 26 25 0.25 -m 26 27 0.25 -m 27 20 0.25 -m 27 34 0.25 -m 27 26 0.25 -m 27 28 0.25 -m 28 21 0.25 -m 28 35 0.25 -m 28 27 0.25 -m 29 22 0.25 -m 29 36 0.25 -m 29 30 0.25 -m 30 23 0.25 -m 30 37 0.25 -m 30 29 0.25 -m 30 31 0.25 -m 31 24 0.25 -m 31 38 0.25 -m 31 30 0.25 -m 31 32 0.25 -m 32 25 0.25 -m 32 39 0.25 -m 32 31 0.25 -m 32 33 0.25 -m 33 26 0.25 -m 33 40 0.25 -m 33 32 0.25 -m 33 34 0.25 -m 34 27 0.25 -m 34 41 0.25 -m 34 33 0.25 -m 34 35 0.25 -m 35 28 0.25 -m 35 42 0.25 -m 35 34 0.25 -m 36 29 0.25 -m 36 43 0.25 -m 36 37 0.25 -m 37 30 0.25 -m 37 44 0.25 -m 37 36 0.25 -m 37 38 0.25 -m 38 31 0.25 -m 38 45 0.25 -m 38 37 0.25 -m 38 39 0.25 -m 39 32 0.25 -m 39 46 0.25 -m 39 38 0.25 -m 39 40 0.25 -m 40 33 0.25 -m 40 47 0.25 -m 40 39 0.25 -m 40 41 0.25 -m 41 34 0.25 -m 41 48 0.25 -m 41 40 0.25 -m 41 42 0.25 -m 42 35 0.25 -m 42 49 0.25 -m 42 41 0.25 -m 43 36 0.25 -m 43 44 0.25 -m 44 37 0.25 -m 44 43 0.25 -m 44 45 0.25 -m 45 38 0.25 -m 45 44 0.25 -m 45 46 0.25 -m 46 39 0.25 -m 46 45 0.25 -m 46 47 0.25 -m 47 40 0.25 -m 47 46 0.25 -m 47 48 0.25 -m 48 41 0.25 -m 48 47 0.25 -m 48 49 0.25 -m 49 42 0.25 -m 49 48 0.25

Figure 2 panel a:

---

ms 2 1000000 -T -L -I 2 2 0 1

ms 2 1000000 -T -L -I 2 2 0 -m 1 2 1 -m 2 1 1 -n 2 1 -ej 2 2 1

ms 2 1000000 -T -L -I 2 2 0 -m 1 2 1 -m 2 1 1 -n 2 1 -ej 2 2 1 -en 2 1 2

Figure 2 panel b:

---

ms 2 2000000 -T -L -I 2 2 0 1

ms 2 1000000 -T -L -I 2 2 0 -ma x 1 1 x -n 2 1 -ej 0.5 1 2

ms 2 1000000 -T -L -I 2 2 0 -ma x 1 1 x -n 2 1 -ej 1 1 2

ms 2 1000000 -T -L -I 2 2 0 -ma x 1 1 x -n 2 1 -ej 2 1 2

ms 2 1000000 -T -L -I 2 2 0 -ma x 1 1 x -n 2 1 -ej 5 1 2

ms 2 1000000 -T -L -I 2 2 0 -ma x 1 1 x -n 2 1 -ej 10 1 2

Figure 2 panel c:

---

ms 2 1000000 -T -L -I 3 2 0 0 1

ms 2 1000000 -T -L -I 3 2 0 0 1 -ej 1 3 2 -ej 3 2 1

ms 2 1000000 -T -L -I 3 0 2 0 1 -ej 1 3 2 -ej 3 2 1

ms 2 1000000 -T -L -I 3 0 0 2 1 -ej 1 3 2 -ej 3 2 1

Figure 2 panel d:

---

ms 2 1000000 -T -L -I 3 2 0 0 1

ms 2 1000000 -T -L -I 3 2 0 0 1 -ej 1.5 3 2 -en 1.5 2 2 -ej 5 2 1 -en 5 1 3

ms 2 1000000 -T -L -I 3 0 2 0 1 -ej 1.5 3 2 -en 1.5 2 2 -ej 5 2 1 -en 5 1 3

ms 2 1000000 -T -L -I 3 0 0 2 1 -ej 1.5 3 2 -en 1.5 2 2 -ej 5 2 1 -en 5 1 3

Figure 3 panel a:

---

ms 2 1000000 -T -L -I 2 2 0 1

ms 2 1000000 -T -L -I 2 2 0 1 -m 2 1 0.1

ms 2 1000000 -T -L -I 2 0 2 1 -m 2 1 0.1

Figure 3 panel b:

---

ms 2 1000000 -T -L -I 3 2 0 0 1

ms 2 1000000 -T -L -I 3 2 0 0 -m 1 2 1 -m 2 1 0.1 -m 2 3 1 -m 3 2 0.1 -m 1 3 1 -m 3 1 0.01

ms 2 1000000 -T -L -I 3 0 2 0 -m 1 2 1 -m 2 1 0.1 -m 2 3 1 -m 3 2 0.1 -m 1 3 1 -m 3 1 0.01

ms 2 1000000 -T -L -I 3 0 0 2 -m 1 2 1 -m 2 1 0.1 -m 2 3 1 -m 3 2 0.1 -m 1 3 1 -m 3 1 0.01

Figure 3 panel c:

---

ms 2 1000000 -T -L -I 2 2 0 1

ms 2 1000000 -T -L -I 2 2 0 1 -n 1 10

ms 2 1000000 -T -L -I 2 0 2 1 -n 1 10

Figure 4 panel a:

---

ms 2 1000000 -T -L -I 2 2 0 1

ms 2 1000000 -T -L -I 2 2 0 1 -m 2 1 0.01

ms 2 1000000 -T -L -I 2 0 2 1 -m 2 1 0.01

Figure 4 panel b:

---

ms 2 1000000 -T -L -I 3 1 1 0 1

ms 2 1000000 -T -L -I 3 1 1 0 -m 1 2 1 -m 2 1 0.1 -m 2 3 1 -m 3 2 0.1 -m 1 3 1 -m 3 1 0.01

ms 2 1000000 -T -L -I 3 1 0 1 -m 1 2 1 -m 2 1 0.1 -m 2 3 1 -m 3 2 0.1 -m 1 3 1 -m 3 1 0.01

ms 2 1000000 -T -L -I 3 0 1 1 -m 1 2 1 -m 2 1 0.1 -m 2 3 1 -m 3 2 0.1 -m 1 3 1 -m 3 1 0.01

---

5 *ms* commands used for the Supplementary figures

For figure S9 panel a the *ms* commands used were:

---

ms 2 1000000 -T -L -I 6 2 0 0 0 0 0 1

ms 2 1000000 -T -L -I 6 2 0 0 0 0 0 -m 1 2 0.1 -m 2 1 0.1 -m 2 3 0.1 -m 3 2 0.1 -m 4 5 0.1 -m 5 4 0.1 -m 5 6 0.1 -m 6 5 0.1 -m 3 4 0.1 -m 4 3 0.1

ms 2 1000000 -T -L -I 6 2 0 0 0 0 0 -m 1 2 1 -m 2 1 1 -m 2 3 1 -m 3 2 1 -m 4 5 1 -m 5 4 1 -m 5 6 1 -m 6 5 1 -m 3 4 1 -m 4 3 1

ms 2 1000000 -T -L -I 6 2 0 0 0 0 0 -m 1 2 5 -m 2 1 5 -m 2 3 5 -m 3 2 5 -m 4 5 5 -m 5 4 5 -m 5 6 5 -m 6 5 5 -m 3 4 5 -m 4 3 5

ms 2 1000000 -T -L -I 6 2 0 0 0 0 0 -m 1 2 10 -m 2 1 10 -m 2 3 10 -m 3 2 10 -m 4 5 10 -m 5 4 10 -m 5 6 10 -m 6 5 10 -m 3 4 10 -m 4 3 10

For figure S9 panel b the *ms* commands used were:

---

ms 2 1000000 -T -L -I 6 2 0 0 0 0 0 1

ms 2 1000000 -T -L -I 9 2 0 0 0 0 0 0 0 0 1

ms 2 1000000 -T -L -I 6 2 0 0 0 0 0 -m 1 2 0.5 -m 2 1 0.5 -m 2 3 0.5 -m 3 2 0.5 -m 4 5 0.5 -m 5 4 0.5 -m 5 6 0.5 -m 6 5 0.5 -m 3 4 0.5 -m 4 3 0.5

ms 2 1000000 -T -L -I 9 2 0 0 0 0 0 0 0 0 -m 1 2 0.5 -m 2 1 0.5 -m 2 3 0.5 -m 3 2 0.5 -m 3 4 0.5 -m 4 3 0.5 -m 4 5 0.5 -m 5 4 0.5 -m 5 6 0.5 -m 6 5 0.5 -m 6 7 0.5 -m 7 6 0.5 -m 7 8 0.5 -m 8 7 0.5 -m 8 9 0.5 -m 9 8 0.5

ms 2 1000000 -T -L -I 6 0 2 0 0 0 0 -m 1 2 0.5 -m 2 1 0.5 -m 2 3 0.5 -m 3 2 0.5 -m 4 5 0.5 -m 5 4 0.5 -m 5 6 0.5 -m 6 5 0.5 -m 3 4 0.5 -m 4 3 0.5

ms 2 1000000 -T -L -I 9 0 2 0 0 0 0 0 0 0 -m 1 2 0.5 -m 2 1 0.5 -m 2 3 0.5 -m 3 2 0.5 -m 3 4 0.5 -m 4 3 0.5 -m 4 5 0.5 -m 5 4 0.5 -m 5 6 0.5 -m 6 5 0.5 -m 6 7 0.5 -m 7 6 0.5 -m 7 8 0.5 -m 8 7 0.5 -m 8 9 0.5 -m 9 8 0.5

ms 2 1000000 -T -L -I 6 0 0 2 0 0 0 -m 1 2 0.5 -m 2 1 0.5 -m 2 3 0.5 -m 3 2 0.5 -m 4 5 0.5 -m 5 4 0.5 -m 5 6 0.5 -m 6 5 0.5 -m 3 4 0.5 -m 4 3 0.5

ms 2 1000000 -T -L -I 9 0 0 2 0 0 0 0 0 0 -m 1 2 0.5 -m 2 1 0.5 -m 2 3 0.5 -m 3 2 0.5 -m 3 4 0.5 -m 4 3 0.5 -m 4 5 0.5 -m 5 4 0.5 -m 5 6 0.5 -m 6 5 0.5 -m 6 7 0.5 -m 7 6 0.5 -m 7 8 0.5 -m 8 7 0.5 -m 8 9 0.5 -m 9 8 0.5

For figure S9 panel c the *ms* commands used were:

---

ms 2 1000000 -T -L -I 9 2 0 0 0 0 0 0 0 0 1

ms 2 1000000 -T -L -I 9 2 0 0 0 0 0 0 0 0 -m 1 4 0.25 -m 1 2 0.25 -m 2 5 0.25 -m 2 1 0.25 -m 2 3 0.25 -m 3 6 0.25 -m 3 2 0.25 -m 4 1 0.25 -m 4 7 0.25 -m 4 5 0.25 -m 5 2 0.25 -m 5 8 0.25 -m 5 4 0.25 -m 5 6 0.25 -m 6 3 0.25 -m 6 9 0.25 -m 6 5 0.25 -m 7 4 0.25 -m 7 8 0.25 -m 8 5 0.25 -m 8 7 0.25 -m 8 9 0.25 -m 9 6 0.25 -m 9 8 0.25

ms 2 1000000 -T -L -I 9 0 2 0 0 0 0 0 0 0 -m 1 4 0.25 -m 1 2 0.25 -m 2 5 0.25 -m 2 1 0.25 -m 2 3 0.25 -m 3 6 0.25 -m 3 2 0.25 -m 4 1 0.25 -m 4 7 0.25 -m 4 5 0.25 -m 5 2 0.25 -m 5 8 0.25 -m 5 4 0.25 -m 5 6 0.25 -m 6 3 0.25 -m 6 9 0.25 -m 6 5 0.25 -m 7 4 0.25 -m 7 8 0.25 -m 8 5 0.25 -m 8 7 0.25 -m 8 9 0.25 -m 9 6 0.25 -m 9 8 0.25

ms 2 1000000 -T -L -I 9 0 0 0 0 2 0 0 0 0 -m 1 4 0.25 -m 1 2 0.25 -m 2 5 0.25 -m 2 1 0.25 -m 2 3 0.25 -m 3 6 0.25 -m 3 2 0.25 -m 4 1 0.25 -m 4 7 0.25 -m 4 5 0.25 -m 5 2 0.25 -m 5 8 0.25 -m 5 4 0.25 -m 5 6 0.25 -m 6 3 0.25 -m 6 9 0.25 -m 6 5 0.25 -m 7 4 0.25 -m 7 8 0.25 -m 8 5 0.25 -m 8 7 0.25 -m 8 9 0.25 -m 9 6 0.25 -m 9 8 0.25

For figure S9 panel d the *ms* commands used were:

---

ms 2 1000000 -T -L -I 25 2 0 0 0 0 0 0 0 0 0 0 0 0 0 0 0 0 0 0 0 0 0 0 0 0 1

ms 2 1000000 -T -L -I 25 2 0 0 0 0 0 0 0 0 0 0 0 0 0 0 0 0 0 0 0 0 0 0 0 0 -m 1 6 0.25 -m 1 2 0.25 -m 2 7 0.25 -m 2 1 0.25 -m 2 3 0.25 -m 3 8 0.25 -m 3 2 0.25 -m 3 4 0.25 -m 4 9 0.25 -m 4 3 0.25 -m 4 5 0.25 -m 5 10 0.25 -m 5 4 0.25 -m 6 1 0.25 -m 6 11 0.25 -m 6 7 0.25 -m 7 2 0.25 -m 7 12 0.25 -m 7 6 0.25 -m 7 8 0.25 -m 8 3 0.25 -m 8 13 0.25 -m 8 7 0.25 -m 8 9 0.25 -m 9 4 0.25 -m 9 14 0.25 -m 9 8 0.25 -m 9 10 0.25 -m 10 5 0.25 -m 10 15 0.25 -m 10 9 0.25 -m 11 6 0.25 -m 11 16 0.25 -m 11 12 0.25 -m 12 7 0.25 -m 12 17 0.25 -m 12 11 0.25 -m 12 13 0.25 -m 13 8 0.25 -m 13 18 0.25 -m 13 12 0.25 -m 13 14 0.25 -m 14 9 0.25 -m 14 19 0.25 -m 14 13 0.25 -m 14 15 0.25 -m 15 10 0.25 -m 15 20 0.25 -m 15 14 0.25 -m 16 11 0.25 -m 16 21 0.25 -m 16 17 0.25 -m 17 12 0.25 -m 17 22 0.25 -m 17 16 0.25 -m 17 18 0.25 -m 18 13 0.25 -m 18 23 0.25 -m 18 17 0.25 -m 18 19 0.25 -m 19 14 0.25 -m 19 24 0.25 -m 19 18 0.25 -m 19 20 0.25 -m 20 15 0.25 -m 20 25 0.25 -m 20 19 0.25 -m 21 16 0.25 -m 21 22 0.25 -m 22 17 0.25 -m 22 21 0.25 -m 22 23 0.25 -m 23 18 0.25 -m 23 22 0.25 -m 23 24 0.25 -m 24 19 0.25 -m 24 23 0.25 -m 24 25 0.25 -m 25 20 0.25 -m 25 24 0.25

ms 2 1000000 -T -L -I 25 0 0 2 0 0 0 0 0 0 0 0 0 0 0 0 0 0 0 0 0 0 0 0 0 0 -m 1 6 0.25 -m 1 2 0.25 -m 2 7 0.25 -m 2 1 0.25 -m 2 3 0.25 -m 3 8 0.25 -m 3 2 0.25 -m 3 4 0.25 -m 4 9 0.25 -m 4 3 0.25 -m 4 5 0.25 -m 5 10 0.25 -m 5 4 0.25 -m 6 1 0.25 -m 6 11 0.25 -m 6 7 0.25 -m 7 2 0.25 -m 7 12 0.25 -m 7 6 0.25 -m 7 8 0.25 -m 8 3 0.25 -m 8 13 0.25 -m 8 7 0.25 -m 8 9 0.25 -m 9 4 0.25 -m 9 14 0.25 -m 9 8 0.25 -m 9 10 0.25 -m 10 5 0.25 -m 10 15 0.25 -m 10 9 0.25 -m 11 6 0.25 -m 11 16 0.25 -m 11 12 0.25 -m 12 7 0.25 -m 12 17 0.25 -m 12 11 0.25 -m 12 13 0.25 -m 13 8 0.25 -m 13 18 0.25 -m 13 12 0.25 -m 13 14 0.25 -m 14 9 0.25 -m 14 19 0.25 -m 14 13 0.25 -m 14 15 0.25 -m 15 10 0.25 -m 15 20 0.25 -m 15 14 0.25 -m 16 11 0.25 -m 16 21 0.25 -m 16 17 0.25 -m 17 12 0.25 -m 17 22 0.25 -m 17 16 0.25 -m 17 18 0.25 -m 18 13 0.25 -m 18 23 0.25 -m 18 17 0.25 -m 18 19 0.25 -m 19 14 0.25 -m 19 24 0.25 -m 19 18 0.25 -m 19 20 0.25 -m 20 15 0.25 -m 20 25 0.25 -m 20 19 0.25 -m 21 16 0.25 -m 21 22 0.25 -m 22 17 0.25 -m 22 21 0.25 -m 22 23 0.25 -m 23 18 0.25 -m 23 22 0.25 -m 23 24 0.25 -m 24 19 0.25 -m 24 23 0.25 -m 24 25 0.25 -m 25 20 0.25 -m 25 24 0.25

ms 2 1000000 -T -L -I 25 0 0 0 0 0 0 0 0 0 0 0 0 2 0 0 0 0 0 0 0 0 0 0 0 0 -m 1 6 0.25 -m 1 2 0.25 -m 2 7 0.25 -m 2 1 0.25 -m 2 3 0.25 -m 3 8 0.25 -m 3 2 0.25 -m 3 4 0.25 -m 4 9 0.25 -m 4 3 0.25 -m 4 5 0.25 -m 5 10 0.25 -m 5 4 0.25 -m 6 1 0.25 -m 6 11 0.25 -m 6 7 0.25 -m 7 2 0.25 -m 7 12 0.25 -m 7 6 0.25 -m 7 8 0.25 -m 8 3 0.25 -m 8 13 0.25 -m 8 7 0.25 -m 8 9 0.25 -m 9 4 0.25 -m 9 14 0.25 -m 9 8 0.25 -m 9 10 0.25 -m 10 5 0.25 -m 10 15 0.25 -m 10 9 0.25 -m 11 6 0.25 -m 11 16 0.25 -m 11 12 0.25 -m 12 7 0.25 -m 12 17 0.25 -m 12 11 0.25 -m 12 13 0.25 -m 13 8 0.25 -m 13 18 0.25 -m 13 12 0.25 -m 13 14 0.25 -m 14 9 0.25 -m 14 19 0.25 -m 14 13 0.25 -m 14 15 0.25 -m 15 10 0.25 -m 15 20 0.25 -m 15 14 0.25 -m 16 11 0.25 -m 16 21 0.25 -m 16 17 0.25 -m 17 12 0.25 -m 17 22 0.25 -m 17 16 0.25 -m 17 18 0.25 -m 18 13 0.25 -m 18 23 0.25 -m 18 17 0.25 -m 18 19 0.25 -m 19 14 0.25 -m 19 24 0.25 -m 19 18 0.25 -m 19 20 0.25 -m 20 15 0.25 -m 20 25 0.25 -m 20 19 0.25 -m 21 16 0.25 -m 21 22 0.25 -m 22 17 0.25 -m 22 21 0.25 -m 22 23 0.25 -m 23 18 0.25 -m 23 22 0.25 -m 23 24 0.25 -m 24 19 0.25 -m 24 23 0.25 -m 24 25 0.25 -m 25 20 0.25 -m 25 24 0.25

For figure S9 panel e the *ms* commands used were:

---

ms 2 1000000 -T -L -I 5 2 0 0 0 0 1

ms 2 1000000 -T -L -I 5 2 0 0 0 0 -m 1 2 0.5 -m 2 1 0.5 -m 2 3 0.5 -m 3 2 0.5 -m 3 4 0.5 -m 4 3 0.5 -m 4 5 0.5 -m 5 4 0.5

ms 2 1000000 -T -L -I 10 2 0 0 0 0 0 0 0 0 0 -m 1 2 0.5 -m 2 1 0.5 -m 2 3 0.5 -m 3 2 0.5 -m 3 4 0.5 -m 4 3 0.5 -m 4 5 0.5 -m 5 4 0.5 -m 5 6 0.5 -m 6 5 0.5 -m 6 7 0.5 -m 7 6 0.5 -m 7 8 0.5 -m 8 7 0.5 -m 8 9 0.5 -m 9 8 0.5 -m 9 10 0.5 -m 10 9 0.5

ms 2 1000000 -T -L -I 15 2 0 0 0 0 0 0 0 0 0 0 0 0 0 0 -m 1 2 0.5 -m 2 1 0.5 -m 2 3 0.5 -m 3 2 0.5 -m 3 4 0.5 -m 4 3 0.5 -m 4 5 0.5 -m 5 4 0.5 -m 5 6 0.5 -m 6 5 0.5 -m 6 7 0.5 -m 7 6 0.5 -m 7 8 0.5 -m 8 7 0.5 -m 8 9 0.5 -m 9 8 0.5 -m 9 10 0.5 -m 10 9 0.5 -m 10 11 0.5 -m 11 10 0.5 -m 11 12 0.5 -m 12 11 0.5 -m 12 13 0.5 -m 13 12 0.5 -m 13 14 0.5 -m 14 13 0.5 -m 14 15 0.5 -m 15 14 0.5

ms 2 1000000 -T -L -I 20 2 0 0 0 0 0 0 0 0 0 0 0 0 0 0 0 0 0 0 0 -m 1 2 0.5 -m 2 1 0.5 -m 2 3 0.5 -m 3 2 0.5 -m 3 4 0.5 -m 4 3 0.5 -m 4 5 0.5 -m 5 4 0.5 -m 5 6 0.5 -m 6 5 0.5 -m 6 7 0.5 -m 7 6 0.5 -m 7 8 0.5 -m 8 7 0.5 -m 8 9 0.5 -m 9 8 0.5 -m 9 10 0.5 -m 10 9 0.5 -m 10 11 0.5 -m 11 10 0.5 -m 11 12 0.5 -m 12 11 0.5 -m 12 13 0.5 -m 13 12 0.5 -m 13 14 0.5 -m 14 13 0.5 -m 14 15 0.5 -m 15 14 0.5 -m 15 16 0.5 -m 16 15 0.5 -m 16 17 0.5 -m 17 16 0.5 -m 17 18 0.5 -m 18 17 0.5 -m 18 19 0.5 -m 19 18 0.5 -m 19 20 0.5 -m 20 19 0.5

For figure S10 the *ms* commands used were:

---

ms 2 1000000 -T -L -I 3 2 0 0 0

ms 2 1000000 -T -L -I 3 2 0 0 0 -ej 1 3 2 -ej 3 2 1

ms 2 1000000 -T -L -I 3 0 2 0 0 -ej 1 3 2 -ej 3 2 1

ms 2 1000000 -T -L -I 3 0 0 2 0 -ej 1 3 2 -ej 3 2 1

For figure S11 panel a the *ms* commands used were:

---

ms 2 1000000 -T -L -I 2 2 0 1

ms 2 1000000 -T -L -I 2 2 0 1 -n 1 10

ms 2 1000000 -T -L -I 2 0 2 1 -n 1 10

ms 2 1000000 -T -L -I 2 2 0 1 -n 1 2

ms 2 1000000 -T -L -I 2 0 2 1 -n 1 2

For figure S11 panel b the *ms* commands used were:

---

ms 2 1000000 -T -L -I 2 2 0 1

ms 2 1000000 -T -L -I 2 2 0 1 -n 1 1

ms 2 1000000 -T -L -I 2 0 2 1 -n 1 10

ms 2 1000000 -T -L -I 2 2 0 1 -n 1 3

ms 2 1000000 -T -L -I 2 0 2 1 -n 1 3

For figure S11 panel c the *ms* commands used were:

---

ms 2 1000000 -T -L -I 2 2 0 1

ms 2 1000000 -T -L -I 2 2 0 1 -n 1 100

ms 2 1000000 -T -L -I 2 0 2 1 -n 1 100

5.1 Figures

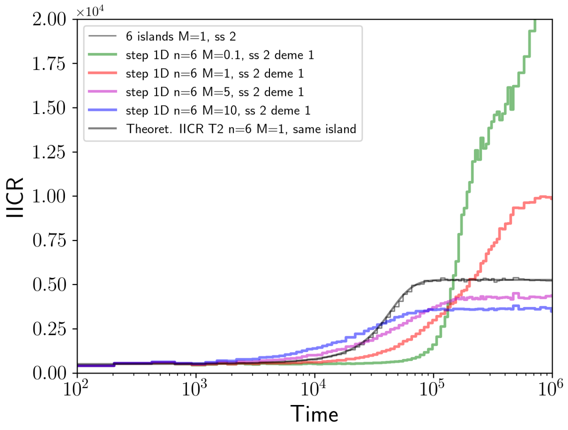

(a) $IICR_s$ 1D, $n = 6$, $M = 0.1; 1; 5; 10$

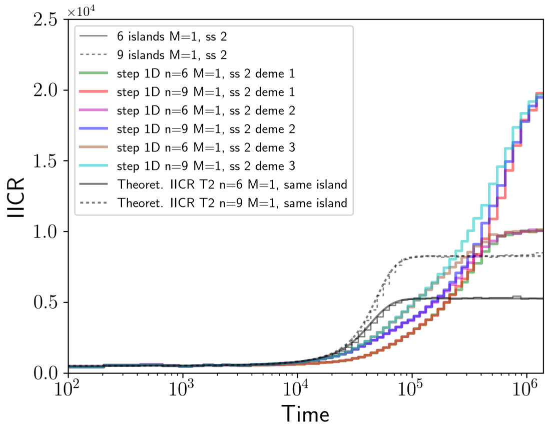

(b) $IICR_s$ 1D, $n = 6; 9$, $M = 1$

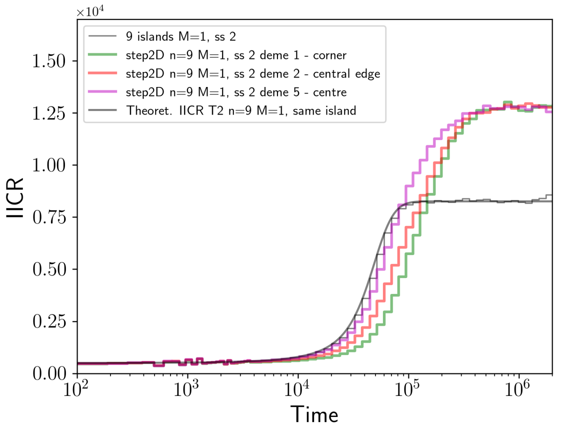

(c) $IICR_s$ 2D, $n = 9$, $M = 1$

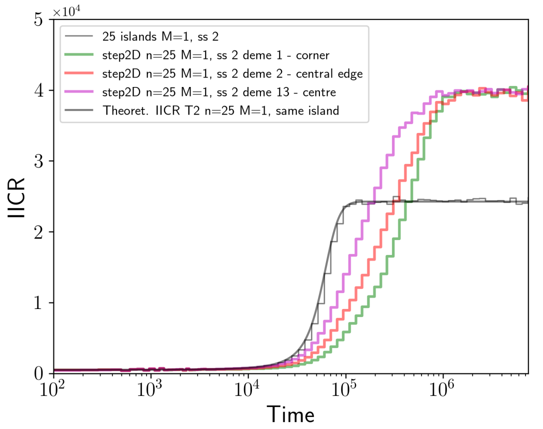

(d) $IICR_s$ 2D, $n = 25$, $M = 1$

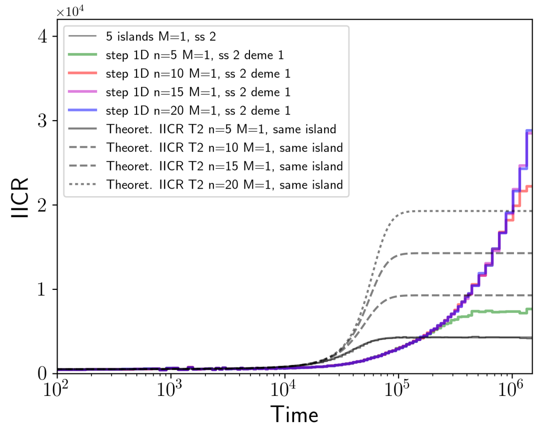

(e) $IICR_s$ 1D, $n = 5; 10; 15; 20$, $M = 1$

Figure S 9: (Continued on the following page.)

Figure S 9: Inferred population size changes ($IICR_s$) for 1D and 2D stepping-stone models with constant size. In the five panels the two genes were sampled in the same deme. Panel (a) shows the result for the 1D stepping-stone model for which we varied M ($M = 0.1$ (green line), $M = 1$ (red line), $M = 5$ (magenta line) and $M = 10$ (blue line)). Panel (b) shows the result for the 1D stepping-stone model for which we varied the number of islands (n) and the deme where the genes were sampled. For a $n = 6$ and $M = 1$ the two genes were sampled in deme 1 (green line), 2 (magenta line) and 3 (sienna line). For a $n = 9$ and $M = 1$ the two genes were sampled in deme 1 (red line), 2 (magenta line) and 3 (light blue line). Panels (c) and (d) show the 2D stepping-stone model results for $n = 9$ and $n = 25$, respectively. In panel (c) the two genes were sampled in deme 1 (corner - green line), 2 (central edge - red line) and 5 (centre - magenta line). In panel (d) the two genes were sampled in deme 1 (corner - green line), 2 (central edge - red line) and 13 (centre - magenta line). Panel (e) shows the result for the 1D stepping-stone model in which we varied n between 5 and 20 ($n = 5$ (green line), $n = 10$ (red line), $n = 15$ (magenta line) and $n = 20$ (blue line)). For comparison we also plotted the results for an n-island model in all panels, using the same simulation approach (black solid and dashed line) and with the same number of islands as the number of demes in the stepping stone models. Finally, we also plotted the theoretical (exact) IICR for the n-island model with the same n and M values (black solid and dashed line) used in the simulations.

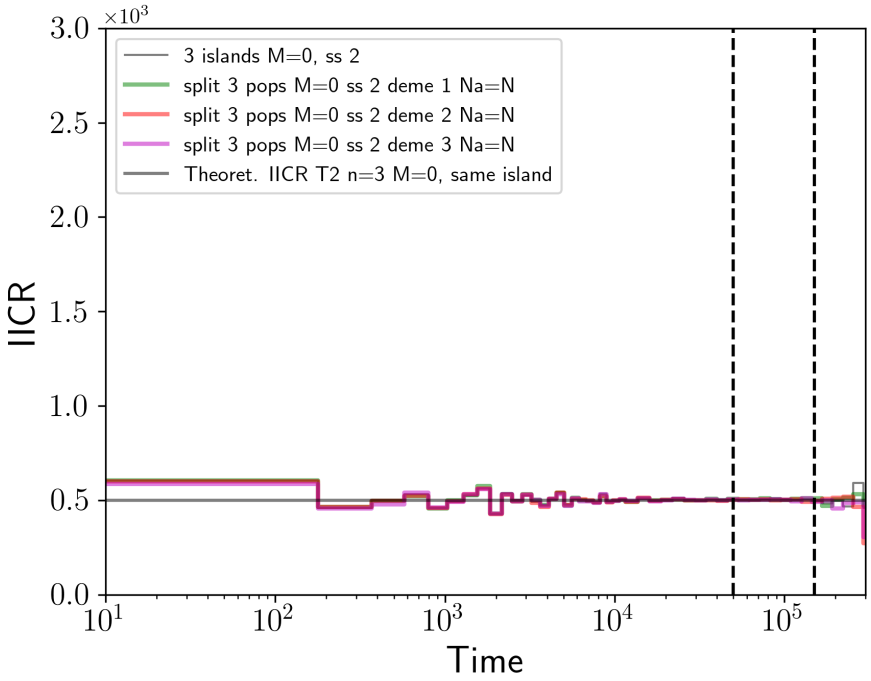

Figure S 10: Inferred population size changes for a split model without gene flow. The two genes were sampled in the same deme. In this case we simulated a splitting model with three populations with an ancestral size N and no gene flow ($M = 0$). At each splitting event there is instantaneous doubling of the population size of the splitting deme. From a coalescent point of view two genes sampled in one deme have thus always "experienced" a constant size N, since there is no gene flow. The $IICR_s$ is thus a horizontal line at a constant "size" N.

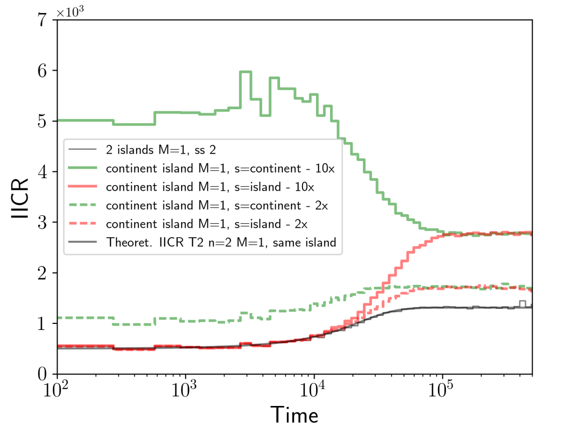

(a) CI model, $r = 1 : 10$ (solid) and $1 : 2$ (dashed)

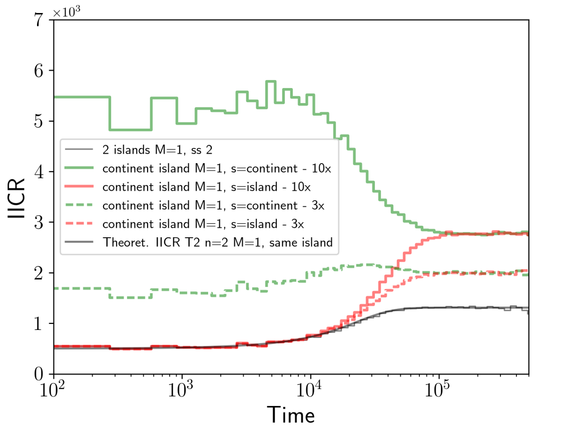

(b) CI model, $r = 1 : 10$ (solid) and $1 : 3$ (dashed)

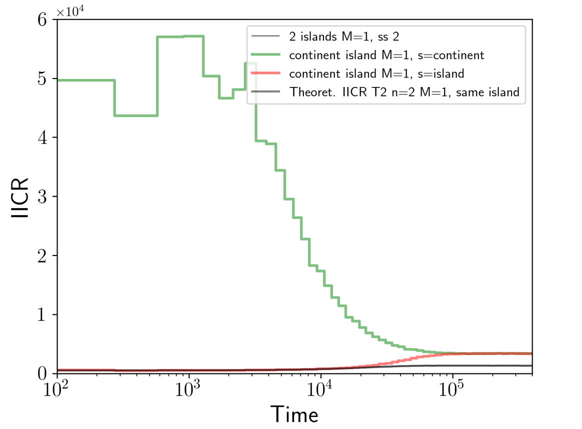

(c) CI model, $r = 1 : 100$

Figure S 11: Continent-island (CI) models with different size ratios (r). In the three panels the two genes were sampled in the same deme. The green line represents the genes sampled in the continent whereas the red line represents the genes sampled in the island. For comparison we also plotted the results for an n-islands model in all panels, using the same simulation approach (black solid line) and with the same number of islands as the number of demes. Finally, we also plotted the theoretical (exact) IICR for the n-islands model for $n = 2$ and $M = 1$ (black solid line).

6 Neanderthal and modern human data

In the Yang *et al.* (2012) study the authors used a series of models to determine whether estimates of admixture between Neanderthal and humans are due to actual admixture events or to ancient structure. They simulated genetic data with *ms* under the two main sets of demographic models, for which they computed a doubly conditioned frequency spectrum ($dcfs$) which they compared to the $dcfs$ of the real data. Their results showed that the model that best fitted their data was the model of recent admixture. We used the *ms* commands of Yang *et al.* (2012) to simulate genetic data under the two demographic models and generate both PSMC (by simulating DNA sequences and using the PSMC method of Li and Durbin (2011)) and $IICR_s$ plots (by simulating T_2 values from the same *ms* commands). We sampled one diploid individual in the "African", "non-African" and "Neanderthal" populations for each of the Yang *et al.* (2012) demographic scenarios. In the main figures of our manuscript we only represented some of the scenarios for ease of representation. Here we present all the combinations of parameter values and corresponding IICR plots. We grouped the scenarios following Yang *et al.* (2012). For the admixture models the authors allowed for bottlenecks that could take place at different times and this is represented below. As a comparison we also represented the PSMC and IICR plots for the model of population size change of Li and Durbin (2011) and the model of population structure of Mazet *et al.* (2016).

Our aim was to determine if the demographic history simulated was compatible with the PSMC plots obtained from humans and Neanderthals. The PSMC method, developed by Li and Durbin (2011), allows us to infer the demographic history of populations. However, this method assumes that populations are panmitic thus generating potential misinterpretations of the PSMC results for structured populations. The IICR plots were used for model exclusion.

The *ms* commands used to generate the PSMC plots (figure S12) are described below.

For the model of recent admixture (bottleneck older than time of admixture):

Neanderthal individual - ms 2 100 -t 65130.39 -r 10973.82 30000000 -I 3 2 0 0 -n 2 100 -n 3 100 -m 3 2 5 -m 2 3 5 -es 0.05 3 0.98 -ej 0.05 4 1 -en 0.1 3 1 -en 0.125 3 100 -ej 0.1125 3 2 -en 0.1150 2 1 -ej 0.3 2 1 -p 8

African individual - ms 2 100 -t 65130.39 -r 10973.82 30000000 -I 3 0 2 0 -n 2 100 -n 3 100 -m 3 2 5 -m 2 3 5 -es 0.05 3 0.98 -ej 0.05 4 1 -en 0.1 3 1 -en 0.125 3 100 -ej 0.1125 3 2 -en 0.1150 2 1 -ej 0.3 2 1 -p 8

Non-African individual - ms 2 100 -t 65130.39 -r 10973.82 30000000 -I 3 0 0 2 -n 2 100 -n 3 100 -m 3 2 5 -m 2 3 5 -es 0.05 3 0.98 -ej 0.05 4 1 -en 0.1 3 1 -en 0.125 3 100 -ej 0.1125 3 2 -en 0.1150 2 1 -ej 0.3 2 1 -p 8

For the model of recent admixture (bottleneck younger than time of admixture):

Neanderthal individual - ms 2 100 -t 65130.39 -r 10973.82 30000000 -I 3 2 0 0 -n 2 100 -n 3 100 -m 3 2 5 -m 2 3 5 -en 0.03 3 1 -en 0.055 3 100 -es 0.05 3 0.98 -ej 0.05 4 1 -ej 0.1125 3 2 -en 0.1150 2 1 -ej 0.3 2 1 -p 8

African individual - ms 2 100 -t 65130.39 -r 10973.82 30000000 -I 3 0 2 0 -n 2 100 -n 3 100 -m 3 2 5 -m 2 3 5 -en 0.03 3 1 -en 0.055 3 100 -es 0.05 3 0.98 -ej 0.05 4 1 -ej 0.1125 3 2 -en 0.1150 2

1 -ej 0.3 2 1 -p 8

Non-African individual - ms 2 100 -t 65130.39 -r 10973.82 30000000 -I 3 0 0 2 -n 2 100 -n 3 100 -m 3 2 5 -m 2 3 5 -en 0.03 3 1 -en 0.055 3 100 -es 0.05 3 0.98 -ej 0.05 4 1 -ej 0.1125 3 2 -en 0.1150 2 1 -ej 0.3 2 1 -p 8

For the model of ancient structure:

Neanderthal individual - ms 2 100 -t 65130.39 -r 10973.82 30000000 -I 3 2 0 0 -n 2 100 -n 3 100 -m 3 2 5 -m 2 3 5 -en 0.03 3 1 -en 0.055 3 100 -em 0.1125 3 2 4 -em 0.1125 2 3 4 -en 0.1150 2 1 -en 0.1150 3 1 -ej 0.3 1 3 -ej 0.6 3 2 -p 8

African individual - ms 2 100 -t 65130.39 -r 10973.82 30000000 -I 3 0 2 0 -n 2 100 -n 3 100 -m 3 2 5 -m 2 3 5 -en 0.03 3 1 -en 0.055 3 100 -em 0.1125 3 2 4 -em 0.1125 2 3 4 -en 0.1150 2 1 -en 0.1150 3 1 -ej 0.3 1 3 -ej 0.6 3 2 -p 8

Non-African individual - ms 2 100 -t 65130.39 -r 10973.82 30000000 -I 3 0 0 2 -n 2 100 -n 3 100 -m 3 2 5 -m 2 3 5 -en 0.03 3 1 -en 0.055 3 100 -em 0.1125 3 2 4 -em 0.1125 2 3 4 -en 0.1150 2 1 -en 0.1150 3 1 -ej 0.3 1 3 -ej 0.6 3 2 -p 8

---

ms command used by Li and Durbin (2011) to simulate genomic data under a population size change model reproducing the human demographic history:

---

ms 2 100 -t 65130.39 -r 10973.82 30000000 -eN 0.0055 0.0832 -eN 0.0089 0.0489 -eN 0.0130 0.0607 -eN 0.0177 0.1072 -eN 0.0233 0.2093 -eN 0.0299 0.3630 -eN 0.0375 0.5041 -eN 0.0465 0.5870 -eN 0.0571 0.6343 -eN 0.0695 0.6138 -eN 0.0840 0.5292 -eN 0.1010 0.4409 -eN 0.1210 0.3749 -eN 0.1444 0.3313 -eN 0.1718 0.3066 -eN 0.2040 0.2952 -eN 0.2418 0.2915 -eN 0.2860 0.2950 -eN 0.3379 0.3103 -eN 0.3988 0.3458 -eN 0.4701 0.4109 -eN 0.5538 0.5048 -eN 0.6520 0.5996 -eN 0.7671 0.6440 -eN 0.9020 0.6178 -eN 1.0603 0.5345 -eN 1.4635 1.7931 -p 8

---

ms command used by Mazet *et al.* (2016) to simulate genomic data under a population structure model reproducing the human demographic history:

---

ms 2 100 -t 1590 -r 318 30000000 -I 10 2 0 0 0 0 0 0 0 0 0 0.55 -eM 4.5 4 -eM 18.0 0.55 -eM 47.5 0.85 -p 8

The *ms* commands used to generate the IICR plots (figure S13) are described below. We used the same commands written above but instead of simulate genomic data, we simulated coalescence times (T_2).

For the model of recent admixture (bottleneck older than time of admixture):

Neanderthal individual - ms 2 1000000 -T -I 3 2 0 0 -n 2 100 -n 3 100 -m 3 2 5 -m 2 3 5 -es 0.05 3 0.98 -ej 0.05 4 1 -en 0.1 3 1 -en 0.125 3 100 -ej 0.1125 3 2 -en 0.1150 2 1 -ej 0.3 2 1

African individual - ms 2 1000000 -T -I 3 0 2 0 -n 2 100 -n 3 100 -m 3 2 5 -m 2 3 5 -es 0.05 3 0.98 -ej 0.05 4 1 -en 0.1 3 1 -en 0.125 3 100 -ej 0.1125 3 2 -en 0.1150 2 1 -ej 0.3 2 1

Non-African individual - ms 2 1000000 -T -I 3 0 0 2 -n 2 100 -n 3 100 -m 3 2 5 -m 2 3 5 -es 0.05 3 0.98 -ej 0.05 4 1 -en 0.1 3 1 -en 0.125 3 100 -ej 0.1125 3 2 -en 0.1150 2 1 -ej 0.3 2 1

For the model of recent admixture (bottleneck younger than time of admixture):

Neanderthal individual - ms 2 1000000 -T -I 3 2 0 0 -n 2 100 -n 3 100 -m 3 2 5 -m 2 3 5 -en 0.03 3 1 -en 0.055 3 100 -es 0.05 3 0.98 -ej 0.05 4 1 -ej 0.1125 3 2 -en 0.1150 2 1 -ej 0.3 2 1

African individual - ms 2 1000000 -T -I 3 0 2 0 -n 2 100 -n 3 100 -m 3 2 5 -m 2 3 5 -en 0.03 3 1 -en 0.055 3 100 -es 0.05 3 0.98 -ej 0.05 4 1 -ej 0.1125 3 2 -en 0.1150 2 1 -ej 0.3 2 1

Non-African individual - ms 2 1000000 -T -I 3 0 0 2 -n 2 100 -n 3 100 -m 3 2 5 -m 2 3 5 -en 0.03 3 1 -en 0.055 3 100 -es 0.05 3 0.98 -ej 0.05 4 1 -ej 0.1125 3 2 -en 0.1150 2 1 -ej 0.3 2 1

For the model of ancient structure:

Neanderthal individual - ms 2 1000000 -T -I 3 2 0 0 -n 2 100 -n 3 100 -m 3 2 5 -m 2 3 5 -en 0.03 3 1 -en 0.055 3 100 -em 0.1125 3 2 4 -em 0.1125 2 3 4 -en 0.1150 2 1 -en 0.1150 3 1 -ej 0.3 1 3 -ej 0.6 3 2

African individual - ms 2 1000000 -T -I 3 0 2 0 -n 2 100 -n 3 100 -m 3 2 5 -m 2 3 5 -en 0.03 3 1 -en 0.055 3 100 -em 0.1125 3 2 4 -em 0.1125 2 3 4 -en 0.1150 2 1 -en 0.1150 3 1 -ej 0.3 1 3 -ej 0.6 3 2

Non-African individual - ms 2 1000000 -T -I 3 0 0 2 -n 2 100 -n 3 100 -m 3 2 5 -m 2 3 5 -en 0.03 3 1 -en 0.055 3 100 -em 0.1125 3 2 4 -em 0.1125 2 3 4 -en 0.1150 2 1 -en 0.1150 3 1 -ej 0.3 1 3 -ej 0.6 3 2

We also tested all the combinations of parameters from Yang *et al.* (2012) study to see if the IICR plots were different. They do not differ significantly, with the exception of the admixture model which can generate one large or tight hump.

The different parameters used in the *ms* commands are described in the following table:
Note - NA = Not Applicable; Deme 1 = Neanderthal population; Deme 2 = African population; Deme 3 = Non-African population

The *ms* commands used to generate the IICR plots (figure S14 to figure S16) are described below.

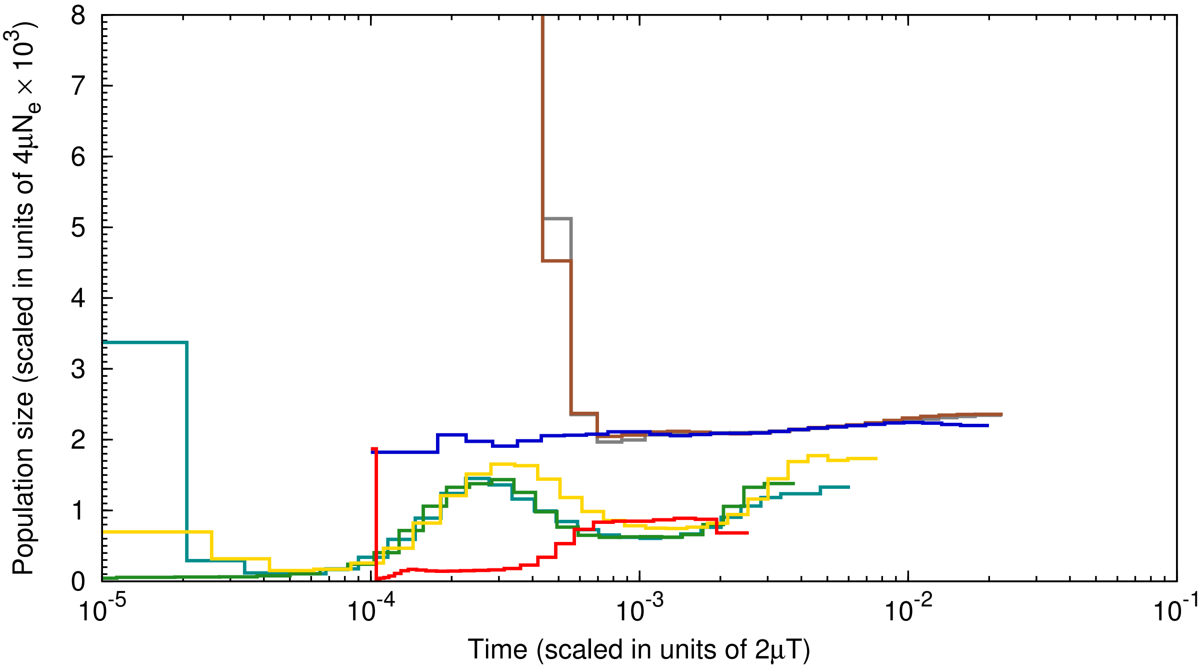

(a) Inferred population history from the Yang *et al.* (2012) (model of recent admixture - bottleneck older than time of admixture), Li and Durbin (2011), Mazet *et al.* (2016) study, a French and a Neanderthal sample

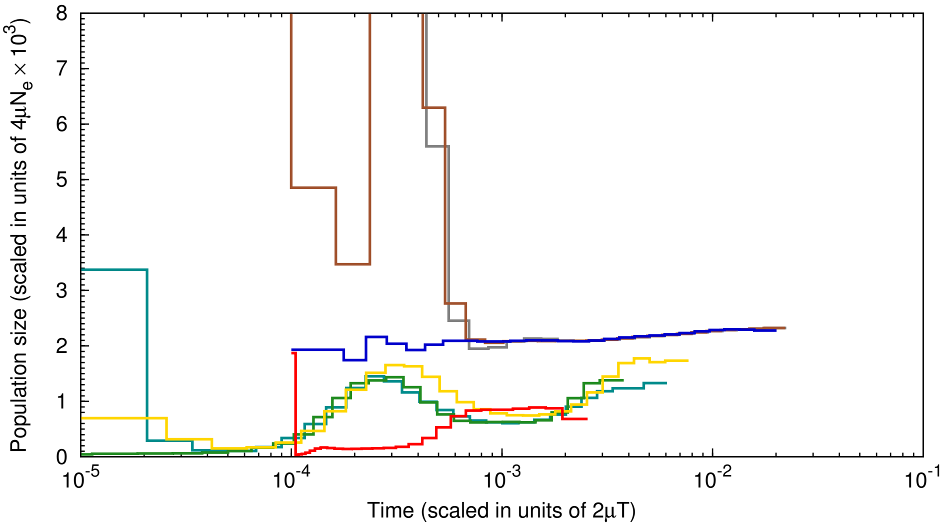

(b) Inferred population history from the Yang *et al.* (2012) (model of recent admixture - bottleneck younger than time of admixture), Li and Durbin (2011), Mazet *et al.* (2016) study, a French and a Neanderthal sample

Figure S 12: (Continued on the following page.)

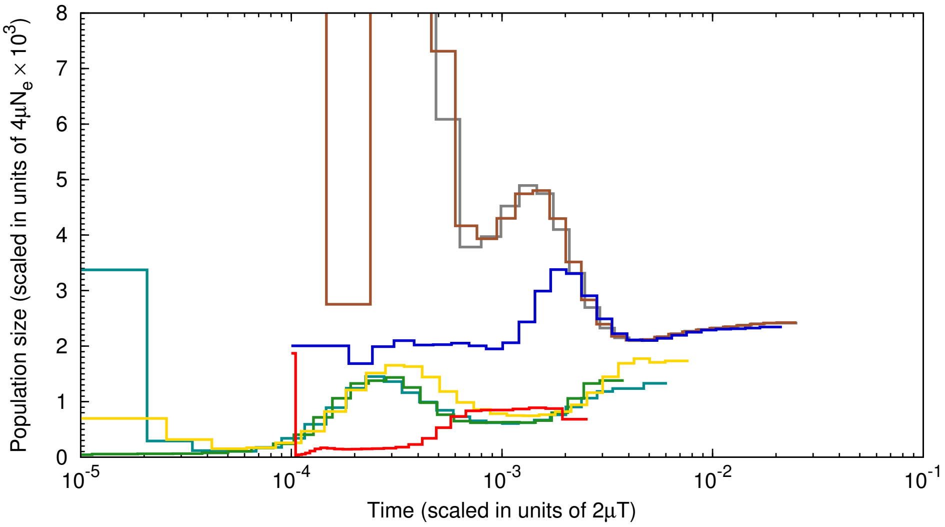

(c) Inferred population history from the Yang *et al.* (2012) (model of ancient structure), Li and Durbin (2011), Mazet *et al.* (2016) study, a French and a Neanderthal sample

Figure S 12: PSMC plots inferred for the human populations. In panel (a) we plot the demographic history for the model of recent admixture (bottleneck older than time of admixture - Yang *et al.* (2012)). Panel (b) we plot the demographic history for the model of recent admixture (bottleneck older than time of admixture - Yang *et al.* (2012)). Panel (c) we plot the demographic history for the model of ancient structure - Yang *et al.* (2012)). For comparison we also plotted (in the four panels) the results obtained in the Li and Durbin (2011) and Mazet *et al.* (2016) study, and the results using genomic data from a French and a Neanderthal individual. Gray line represents an African individual, brown line represents a non-African individual, dark blue line represents a Neanderthal individual, light blue line represents Li and Durbin (2011) simulated data, green line represents Mazet *et al.* (2016) simulated data, yellow line represents a French individual and red line represents a Neanderthal individual.

Model	Admixture rate (f)	Bottleneck time (t_b)	Ongoing gene flow ($4Nm$)	Ancient migration rate ($4Nm_a$)
Recent admixture (bottleneck older than time of admixture)	0.02; 0.03; 0.05; 0.1	1	0; 1; 5	NA
Recent admixture (bottleneck younger than time of admixture)	0.02; 0.03; 0.05; 0.1	0.03	0; 1; 5	NA
Ancient structure	NA	0.03; 1	0; 1; 5	0; 2; 4; 6; 8; 10

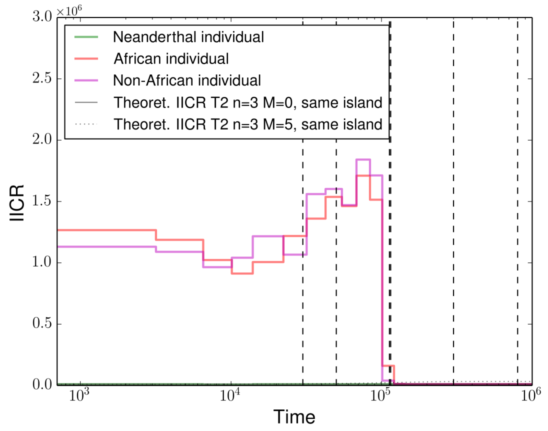

(a) IICR plots for the model of recent admixture (bottleneck older than time of admixture) of Yang *et al.* (2012)

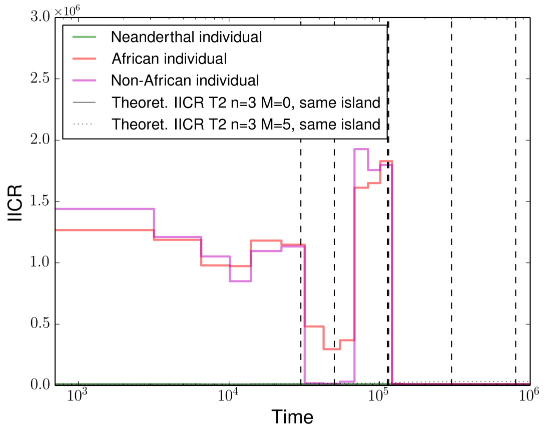

(b) IICR plots for the model of recent admixture (bottleneck younger than time of admixture) of Yang *et al.* (2012)

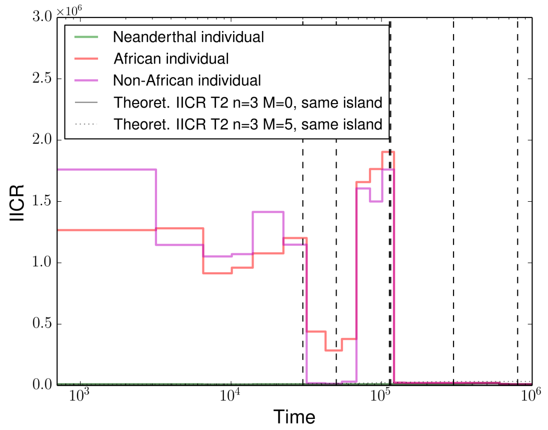

(c) IICR plots for the model of ancient structure of Yang *et al.* (2012)

Figure S 13: IICR plots for the models of admixture and ancient structure of Yang *et al.* (2012). The IICR plots represented in these figures were obtained using *ms* commands of Yang *et al.* (2012) for the admixture and for the ancient structure models. The two genes were sampled in the same deme. Green line represents the demographic history for the Neanderthal individual. Red line represents the demographic history for the African individual. Magenta line represents the demographic history for the non-African individual. We also plotted the theoretical (exact) IICR for the n-island model for $n = 3$ and $M = 0$ (black solid line) and $M = 5$ (black dashed line).

For the model of recent admixture (bottleneck older than time of admixture):

---

ms 2 1000000 -T -I 3 2 0 0 -n 2 100 -n 3 100 -m 3 2 $4Nm$ -m 2 3 $4Nm$ -es 0.05 3 1-f -ej 0.05 4 1 -en t_b 3 1 -en t_b+0.025 3 100 -ej 0.1125 3 2 -en 0.1150 2 1 -ej 0.3 2 1

For the model of recent admixture (bottleneck younger than time of admixture):

---

ms 2 1000000 -T -I 3 2 0 0 -n 2 100 -n 3 100 -m 3 2 $4Nm$ -m 2 3 $4Nm$ -en t_b 3 1 -en t_b+0.025 3 100 -es 0.05 3 1-f -ej 0.05 4 1 -ej 0.1125 3 2 -en 0.1150 2 1 -ej 0.3 2 1

For the model of ancient structure:

---

ms 2 1000000 -T -I 3 2 0 0 -n 2 100 -n 3 100 -m 3 2 $4Nm$ -m 2 3 $4Nm$ -en t_b 3 1 -en t_b+0.025 3 100 -em 0.1125 3 2 $4Nm_a$ -em 0.1125 2 3 $4Nm_a$ -en 0.1150 2 1 -en 0.1150 3 1 -ej 0.3 1 3 -ej 0.6 3 2

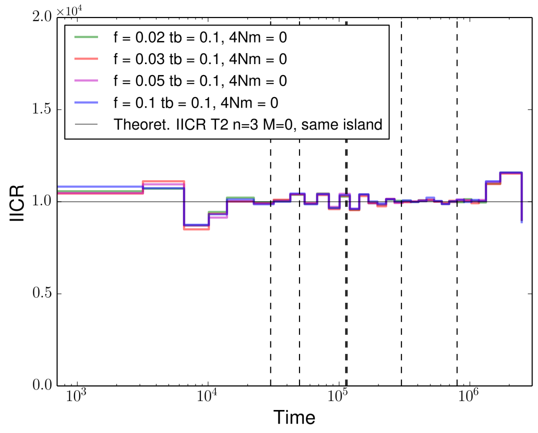

(a) IICR plots Neanderthal individual. $4Nm$=0

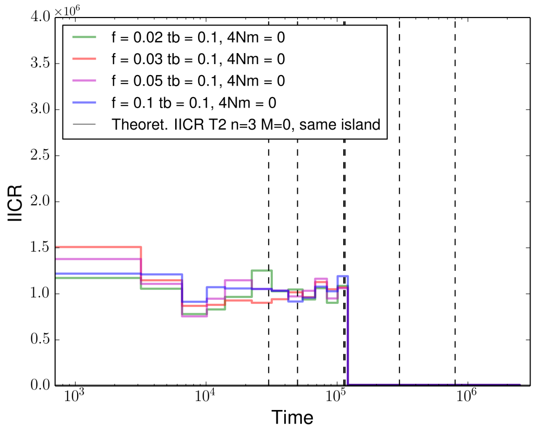

(b) IICR plots African individual. $4Nm$=0

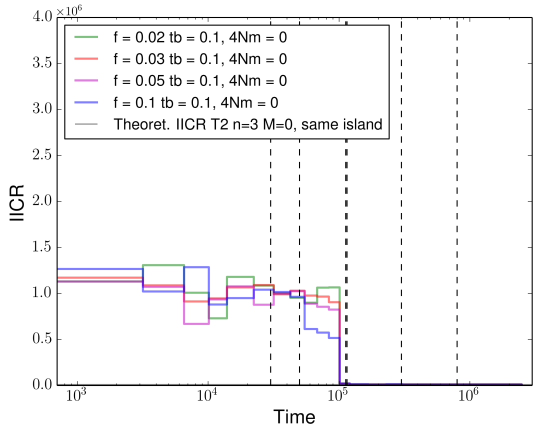

(c) IICR plots non-African individual. $4Nm$=0

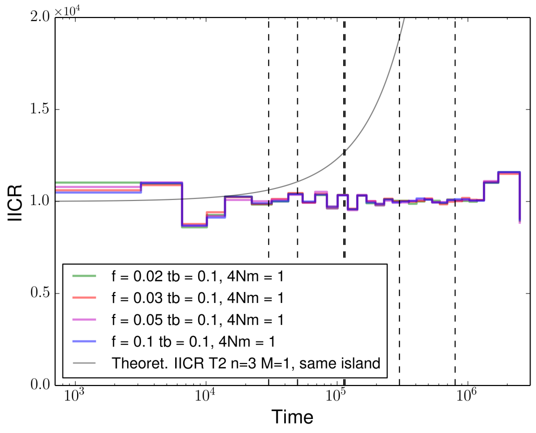

(d) IICR plots Neanderthal individual. $4Nm$=1

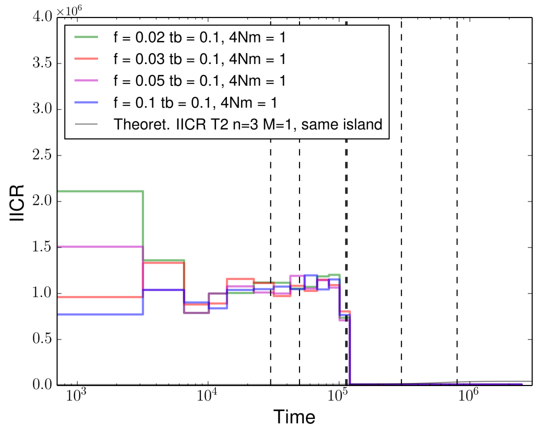

(e) IICR plots African individual. $4Nm$=1

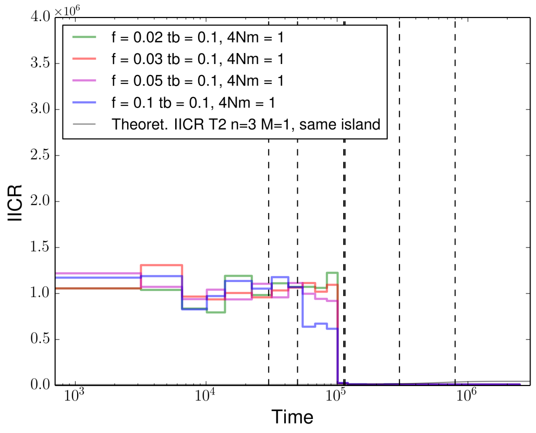

(f) IICR plots non-African individual. $4Nm$=1

Figure S 14: (Continued on the following page.)

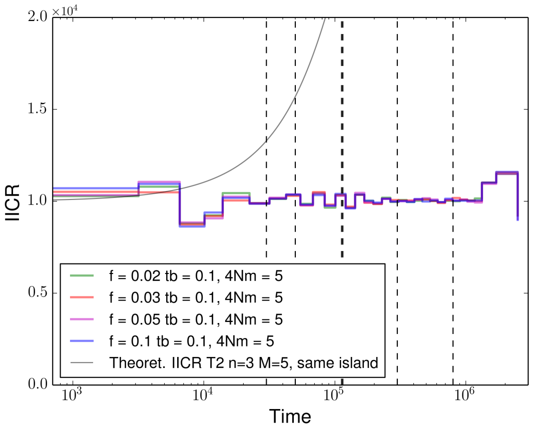

(g) IICR plots Neanderthal individual. $4Nm{=}5$

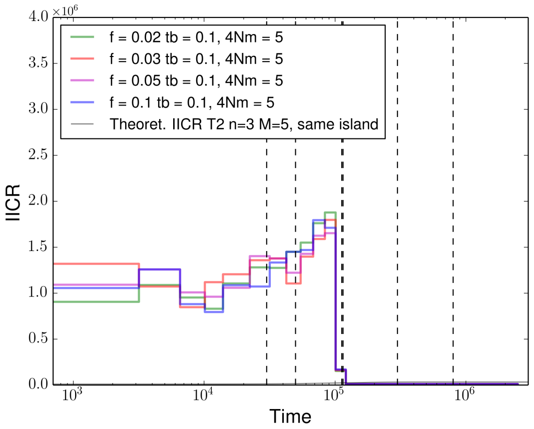

(h) IICR plots African individual. $4Nm{=}5$

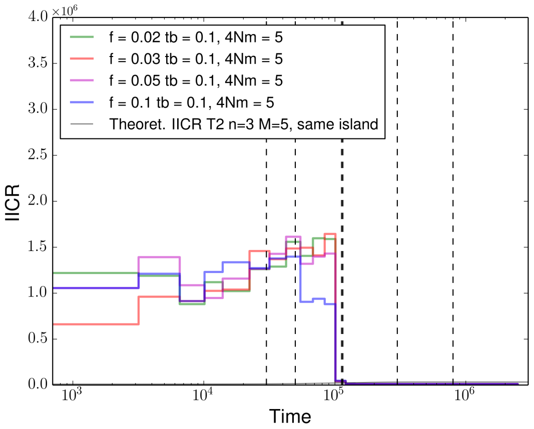

(i) IICR plots non-African individual. $4Nm{=}5$

Figure S 14: IICR plots for the models of recent admixture (bottleneck older than time of admixture) of Yang *et al.* (2012) using all the parameters tested in the article. The IICR plots represented in these figures were obtained using *ms* commands of Yang *et al.* (2012) for the admixture model. The two genes were sampled in the same deme. The bottleneck time is 0.1. In all the panels we used four different colors to distinguish the admixture rate. Green line is for an admixture rate of 0.02. Red line is for an admixture rate of 0.03. Magenta line is for an admixture rate of 0.05. Blue line is for an admixture rate of 0.1. We also plotted the theoretical (exact) IICR for the n-island model for $n = 3$ and $M = 0$; 1; or 5 (black solid line).

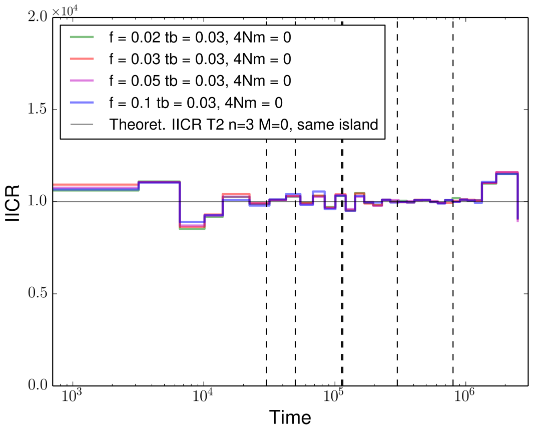

(a) IICR plots Neanderthal individual. $4Nm$=0

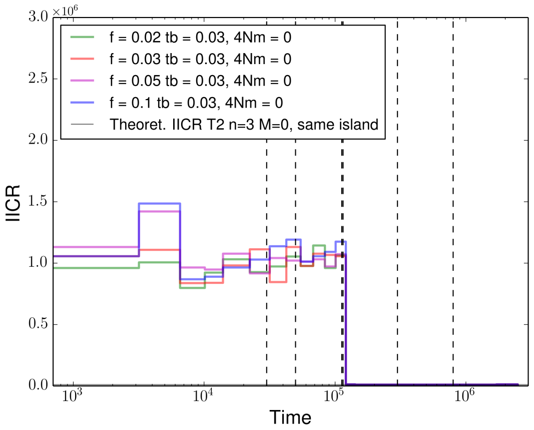

(b) IICR plots African individual. $4Nm$=0

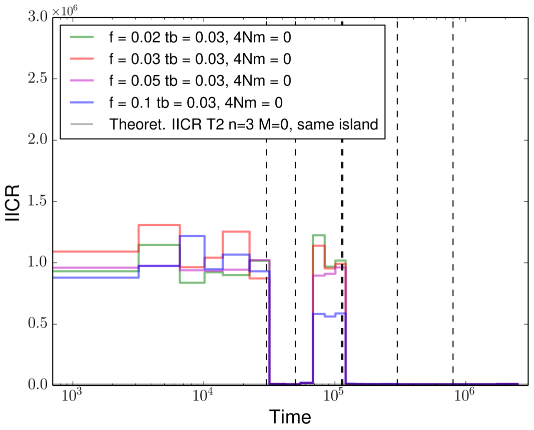

(c) IICR plots non-African individual. $4Nm$=0

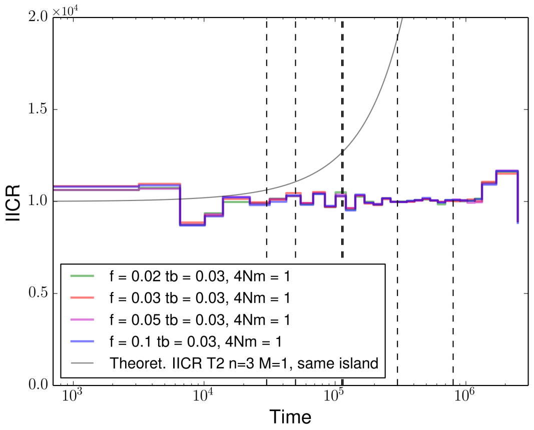

(d) IICR plots Neanderthal individual. $4Nm$=1

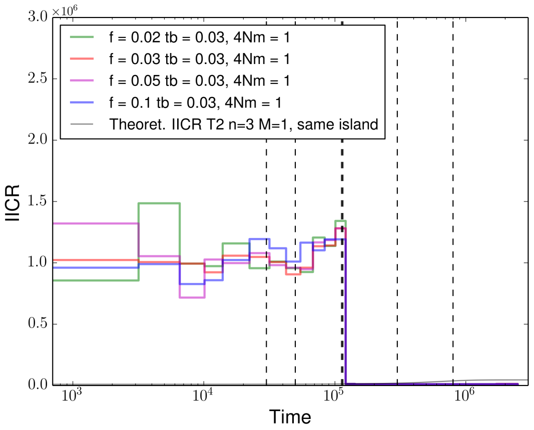

(e) IICR plots African individual. $4Nm$=1

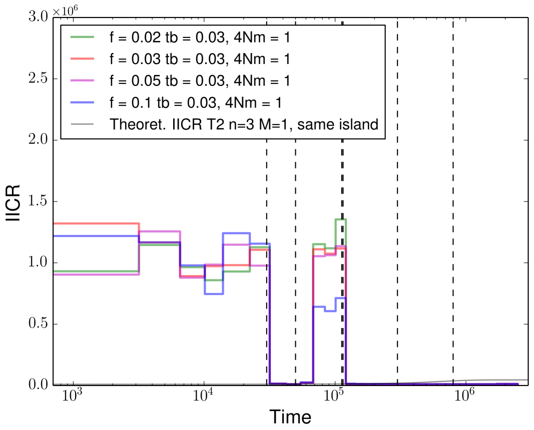

(f) IICR plots non-African individual. $4Nm$=1

Figure S 15: (Continued on the following page.)

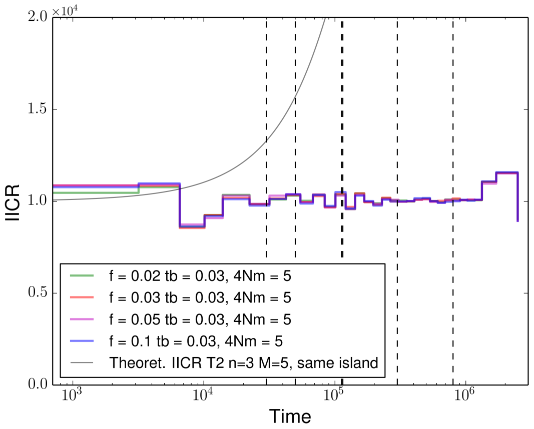

(g) IICR plots Neanderthal individual. $4Nm$=5

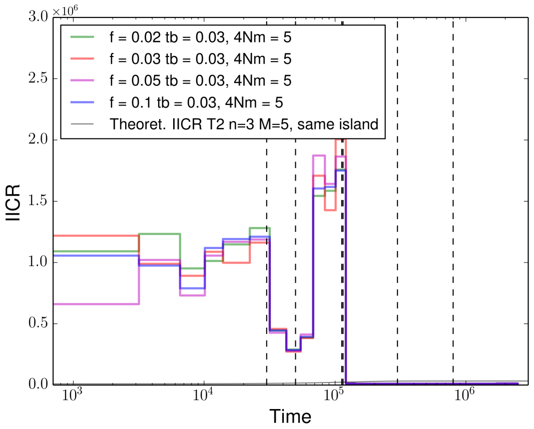

(h) IICR plots African individual. $4Nm$=5

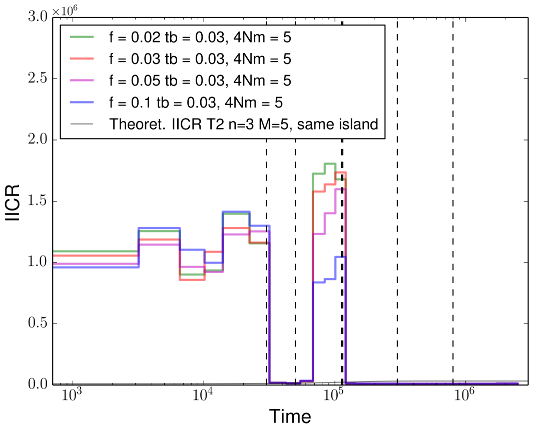

(i) IICR plots non-African individual. $4Nm$=5

Figure S 15: IICR plots for the models of recent admixture (bottleneck younger than time of admixture) of Yang *et al.* (2012) using all the parameters tested in the article. The IICR plots represented in these figures were obtained using *ms* commands of Yang *et al.* (2012) for the admixture model. The two genes were sampled in the same deme. The bottleneck time is 0.03. In all the panels we used four different colors to distinguish the admixture rate. Green line is for an admixture rate of 0.02. Red line is for an admixture rate of 0.03. Magenta line is for an admixture rate of 0.05. Blue line is for an admixture rate of 0.1. We also plotted the theoretical (exact) IICR for the n-island model for n = 3 and M = 0; 1; or 5 (black solid line).

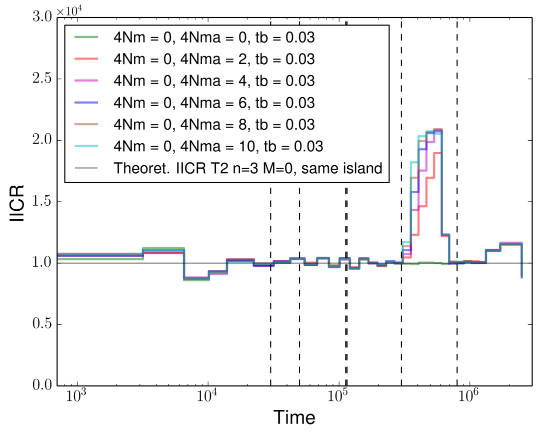

(a) IICR plots Neanderthal individual. $4Nm$=0; t_b=0.03

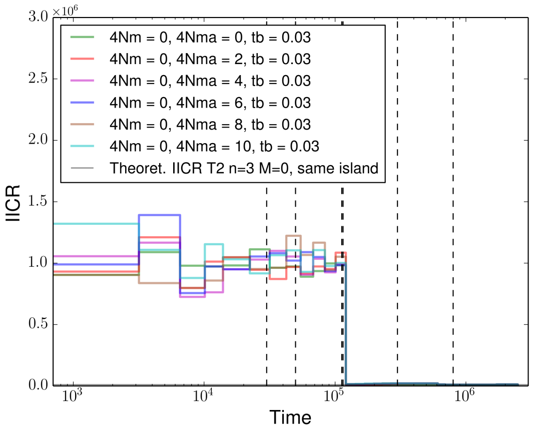

(b) IICR plots African individual. $4Nm$=0; t_b=0.03

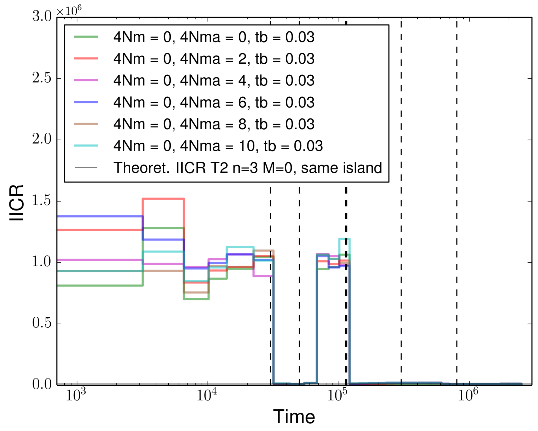

(c) IICR plots non-African individual. $4Nm$=0; t_b=0.03

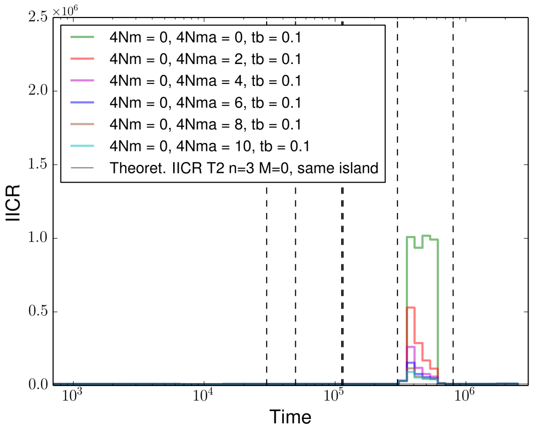

(d) IICR plots Neanderthal individual. $4Nm$=0; t_b=0.1

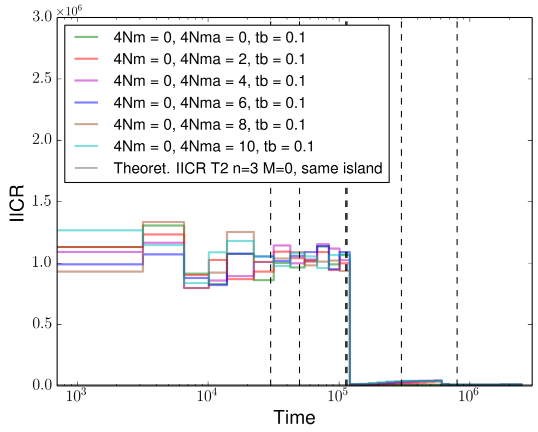

(e) IICR plots African individual. $4Nm$=0; t_b=0.1

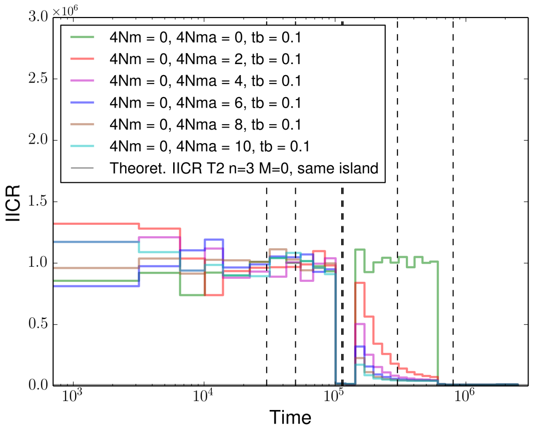

(f) IICR plots non-African individual. $4Nm$=0; t_b=0.1

Figure S 16: (Continued on the following page.)

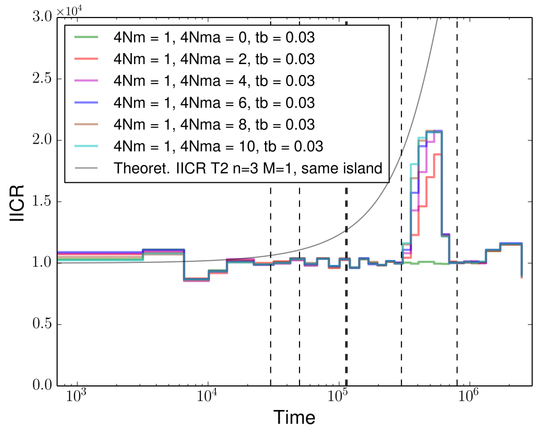

(g) IICR plots Neanderthal individual. $4Nm$=1; t_b=0.03

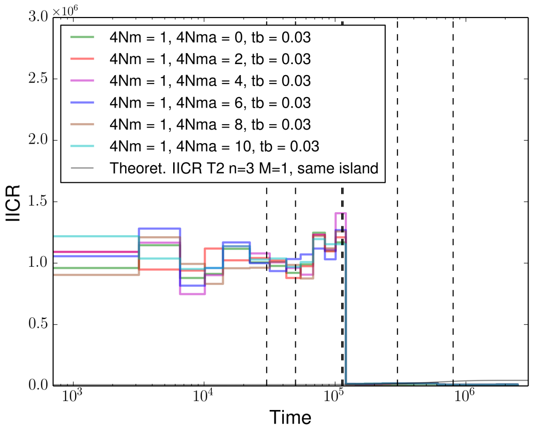

(h) IICR plots African individual. $4Nm$=1; t_b=0.03

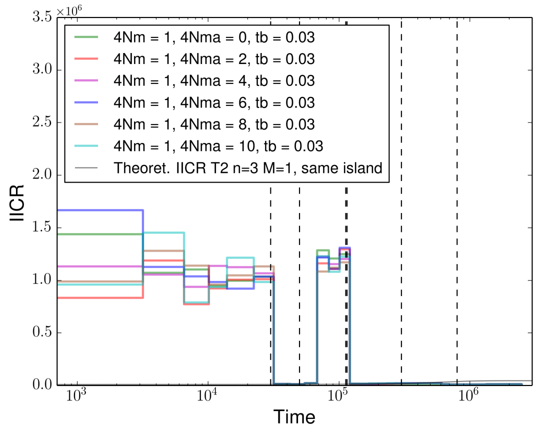

(i) IICR plots non-African individual. $4Nm$=1; t_b=0.03

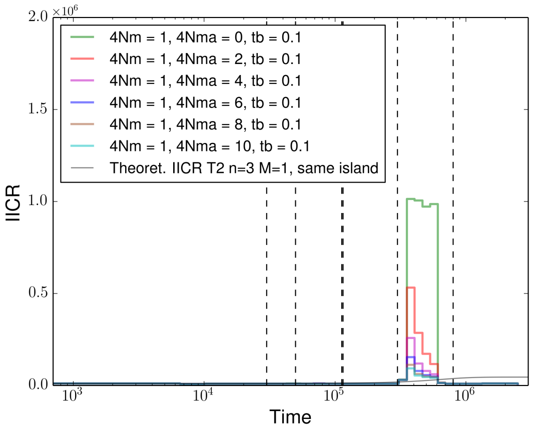

(j) IICR plots Neanderthal individual. $4Nm$=1; t_b=0.1

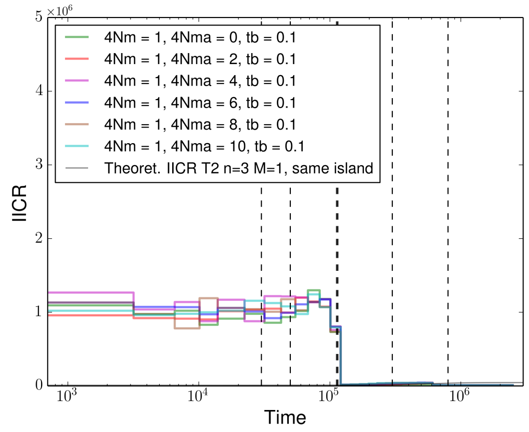

(k) IICR plots African individual. $4Nm$=1; t_b=0.1

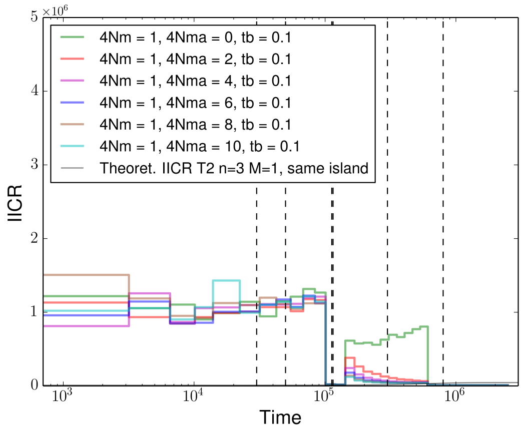

(l) IICR plots non-African individual. $4Nm$=1; t_b=0.1

Figure S 16: (Continued on the following page.)

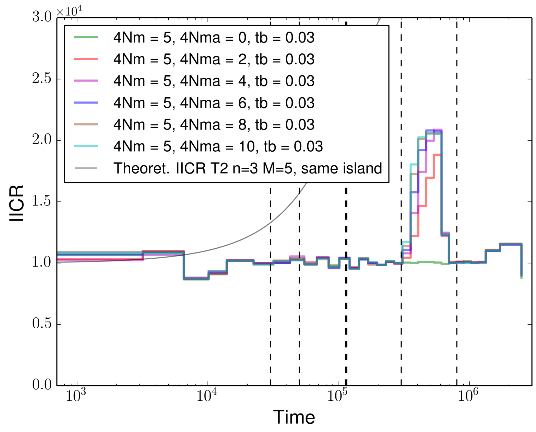

(m) IICR plots Neanderthal individual. $4Nm$=5; t_b=0.03

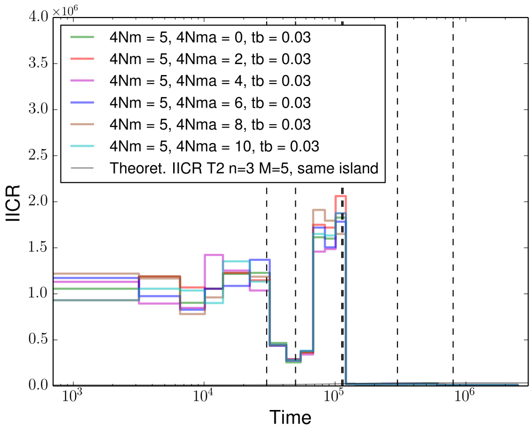

(n) IICR plots African individual. $4Nm$=5; t_b=0.03

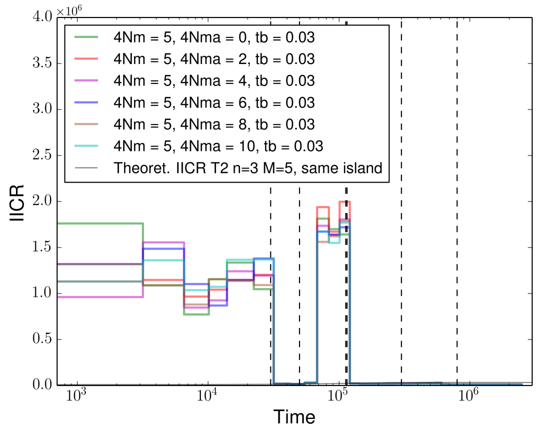

(o) IICR plots non-African individual. $4Nm$=5; t_b=0.03

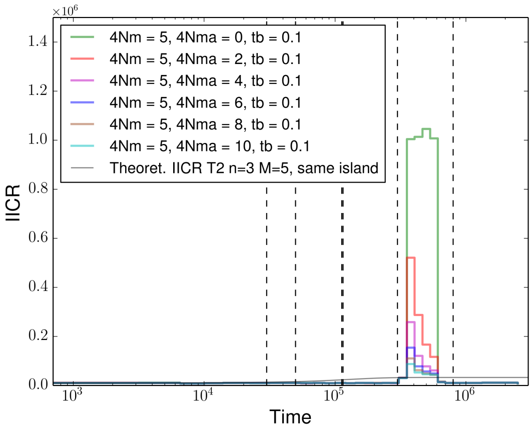

(p) IICR plots Neanderthal individual. $4Nm$=5; t_b=0.1

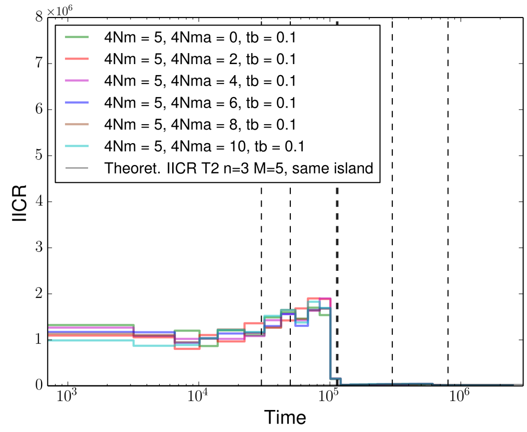

(q) IICR plots African individual. $4Nm$=5; t_b=0.1

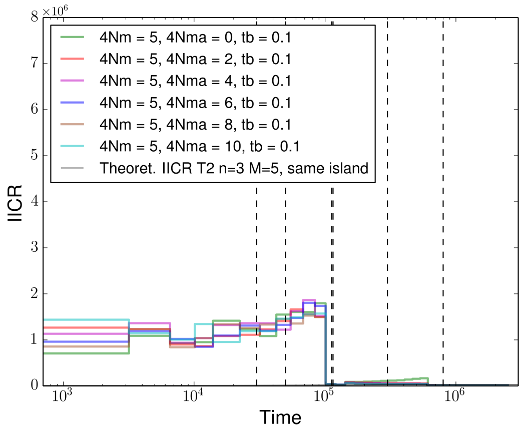

(r) IICR plots non-African individual. $4Nm$=5; t_b=0.1

Figure S 16: (Continued on the following page.)

Figure S 16: IICR plots for the models of ancient structure of Yang *et al.* (2012) using all the parameters tested in the article. The IICR plots represented in these figures were obtained using *ms* commands of Yang *et al.* (2012) for the ancient structure models. The two genes were sampled in the same deme. In all the panels we used six different colors to distinguish the ancient migration rate. Green line is for an ancient migration rate of 0. Red line is for ancient migration rate of 2. Magenta line is for an ancient migration rate of 4. Blue line is for an ancient migration rate of 6. Sienna line is for an ancient migration rate of 8. Cyan line is for an ancient migration rate of 10. We also plotted the theoretical (exact) IICR for the n-island model for $n = 3$ and $M = 0$; 1; or 5 (black solid line).

References

Beaumont M (2004). Recent developments in genetic data analysis: what can they tell us about human demographic history? *Heredity* **92**: 365–379.

Chikhi L, Sousa VC, Luisi P, Goossens B, Beaumont MA (2010). The confounding effects of population structure, genetic diversity and the sampling scheme on the detection and quantification of population size changes. *Genetics* **186**: 983–995.

Heller R, Chikhi L, Siegismund HR (2013). The confounding effect of population structure on Bayesian skyline plot inferences of demographic history. *PLoS One* **8**: e62992.

Hudson RR (2002). Generating samples under a Wright–Fisher neutral model of genetic variation. *Bioinformatics* **18**: 337–338. **URL:** *http://bioinformatics.oxfordjournals.org/content/18/2/337.abstract*

Kimura M, Weiss GH (1964). The stepping stone model of population structure and the decrease of genetic correlation with distance. *Genetics* **49**: 561.

Li H, Durbin R (2011). Inference of human population history from individual whole-genome sequences. *Nature* **475**: 493–496.

Mazet O, Rodriguez W, Grusea S, Boitard S, Chikhi L (2016). On the importance of being structured: instantaneous coalescence rates and human evolution—lessons for ancestral population size inference&quest. *Heredity* **116**: 362–371.

Paz-Vinas I, Quéméré E, Chikhi L, Loot G, Blanchet S (2013). The demographic history of populations experiencing asymmetric gene flow: combining simulated and empirical data. *Molecular Ecology* **22**: 3279–3291.

Städler T, Haubold B, Merino C, Stephan W, Pfaffelhuber P (2009). The impact of sampling schemes on the site frequency spectrum in nonequilibrium subdivided populations. *Genetics* **182**: 205–216.

Wakeley J (1999). Nonequilibrium migration in human history. *Genetics* **153**: 1863–1871.

Yang MA, Malaspinas AS, Durand EY, Slatkin M (2012). Ancient structure in Africa unlikely to explain Neanderthal and non-African genetic similarity. *Molecular biology and evolution* **29**: 2987–2995.
